# “Instead of Building More Buildings, They Should Plant More Trees”, a Photovoice Study of Determinants of Happiness and Sadness Among East London Adolescents

**DOI:** 10.1177/10497323241291667

**Published:** 2024-11-14

**Authors:** Madison Stephens, Nargis Rahmanfard, Maev Conneely, Victoria Bird, Alec Knight, Paul Heritage, Laiba Waseem, Sopnil Nath, Ariba Ansar, Rida Choudhury, Holly Larkin, Wahaj Ali, Mariam Lassoued, Lakshana Vasanthakumar, Meagan Jade Sanchez, Ali Ullah, James Richard Kiernan, Roxanne De Padua-Johnson, Amsika Kandasamy

**Affiliations:** 1 Unit for Social and Community Psychiatry, WHO Collaborating Centre, Wolfson Institute of Population Health, 4617 Queen Mary University of London, London, UK; 2 Division of Psychiatry, 4919 University College London, London, UK; 3 Centre for Education, Faculty of Life Sciences and Medicine, King’s College London, London, UK; 4 People’s Palace Projects, School of English and Drama, 4617 Queen Mary University of London, London, UK; 5 Wolfson Institute of Population Health, 4617 Queen Mary University of London, London, UK; 6 Future Leaders, London, UK

**Keywords:** Photovoice, adolescents, mental health, social determinants, East London, happiness, sadness

## Abstract

Globally, mental health problems in adolescents, alongside associated morbidity and mortality, have never been higher. Local living, working and environmental conditions, socio-economics, and intra-individual and inter-individual processes impact mental health. The risk of developing mental health problems is higher in certain areas, including East London. However, limited research explores East London adolescents’ experiences of mental health. An in-depth and locally situated understanding of determinants shaping East London adolescents’ happiness and sadness is needed. This study used Photovoice, a qualitative method within a community-based participatory research methodology, to generate photographic and textual data, which was analyzed using reflexive thematic analysis. This method allows participants to be part of knowledge production and authors to present the data. Our findings underscore the bidirectional interplay between environmental factors and adolescents’ happiness and sadness. Gratitude for nature was described as increasing happiness: adolescents connected to nature to memories, appreciation, and leisure opportunities. Adolescents were concerned about the fragility of nature in response to urban development. The urban environment was perceived as imposing, inspiring, and offering therapeutic benefits blighted by pollution. Beautiful areas were described as paradisical and lacking, revealing urban development and economic productivity disparities. Our research documents the voices of an under-researched group, revealing novel insights while empowering adolescents as co-producers of mental health research. This study indicates participatory research is valuable for granting adolescents autonomy and addressing misrepresentation. The findings implicate multiple stakeholders, including “Health in All Policies.” By deepening our understanding of adolescent mental health in East London, our study can be leveraged to bolster the effectiveness and relevance of interventions for East London adolescents.

## Introduction

Mental health encompasses uncomfortable feelings such as sadness (Mental Health Foundation, n.d.), which form the human experience (Galderisi et al., 2017) and shape individuals’ health. Mental health problems adversely impact happiness (Almadani & Alwesmi, 2023). Adolescents are susceptible to mental health problems (Blakemore, 2019), accounting for 45% of the global disease burden in this population (Osborn et al., 2020). The burden of mental health problems in adolescents has implications for adolescent mortality, with a leading cause of death among adolescents being suicide (World Health Organisation [WHO], 2021). Prevalence and onset of mental health problems among adolescents have been theorized on and associated with neurodevelopmental and hormonal changes (Lockhart et al., 2018) and personal, academic, and social pressures (Acharya et al., 2018). The risk of mental health problems in adolescents appears to be heightened by certain factors, including inequality (Yu, 2018), economic deprivation (Deighton et al., 2019), and living in urban environments (Shattuck, 2019).

Recent research indicates that determinants within East London, which has high rates of mental health problems compared to other boroughs (Tower Hamlets, 2018), affect the happiness of adolescents residing in the region. For instance, Smith and colleagues (2015) noted a correlation between a lack of neighborhood safety among East London adolescents and depression. Nevertheless, adolescents and populations living in East London remain underrepresented in mental health research (Mawn et al., 2016; University College London, 2022).

**Figure 1.**
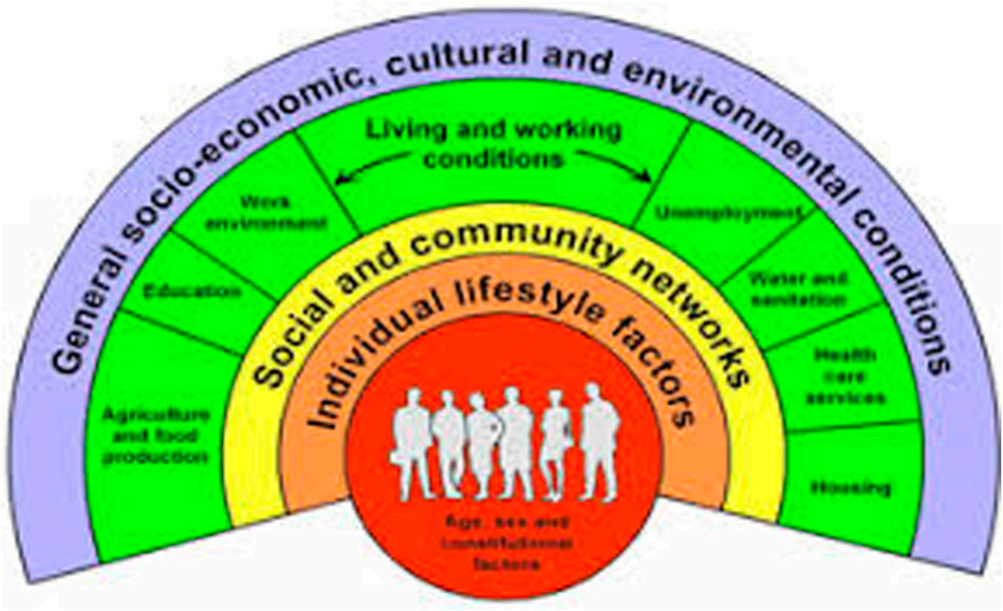
The Dahlgren & Whitehead (1991) model of health determinants.

Understanding how social determinants influence the experiences of mental health of adolescents living in East London is needed. Dahlgren & Whitehead’s (1991) theoretical framework of social determinants (Figure 1, left) recognizes how factors, including environmental conditions and social networks, influence health. Adopting a social determinants perspective of adolescent mental health is relevant, considering mental health problems are influenced by social determinants (WHO, 2014), which are thought to have the most effect on health compared to biological factors (Centres for Disease Control and Prevention, 2022).

Examining social determinants of mental health alongside adolescents living in East London would add to a paucity of evidence and better represent East London adolescents in mental health research. Our approach addresses this gap and advocates for adolescents’ ownership of the mental health discourse concerning them. Conducting this research is crucial, as the findings will likely differ from those of more widely represented populations and locations. Therefore, the significance of this study lies in the potential insights gained from comparing the experiences of East London adolescents with those from different age groups and regions.

Our study could be useful in informing locally specific interventions whose success depends on contextual knowledge (Rogers et al., 2020). Moreover, such research is important in light of increasing urbanization and inequality, a leading health challenge (WHO, 2015) influencing mental health (Pelgrims et al., 2021). The production of local-situated knowledge could inform the design and delivery of local health interventions.

Photovoice combines photography and narrative to gain a deep understanding of group experiences. Photovoice is fun (Wass & Safari, 2020) and emancipating (Schnittker, 2013) and produces rich and nuanced insights (Guell & Ogilvie, 2015), extending beyond those obtained by traditional methods (Levin et al., 2007). Photovoice is helpful in mental health research involving adolescents (Vélez-Grau, 2019) for several reasons. For instance, Photovoice is a method that allows participants to lead, encourages honesty, and benefits individuals’ mental health by providing opportunities for learning, acceptance, and support (Werremeyer et al., 2020). Photovoice can also challenge negative mental health stereotypes (Flanagan et al., 2016). Despite its potential benefits, no adolescent mental health research using Photovoice has been published in England, possibly reflecting the infancy of Photovoice in this field (Stephens et al., 2023).

The lack of evidence helped inform our research question: “How do things and places influence East London adolescents’ perceptions and experiences of happiness and sadness?” This ensured adolescents understood our research aim. The choice of language thus flowed from the philosophical underpinning of the Photovoice method, which aims to empower and includes using language that was not preferred by adolescents, which would have done the opposite.

## Methods

### Terminology and Conceptual Framework

We used the words “happiness” and “sadness” in our research question to honor the suggestions made by adolescents co-designing our research. Adolescents co-designing our research deemed happiness and sadness more understandable than “mental health,” which varies across cultures and people (Galderisi et al., 2017). Happiness and sadness are fundamental, universal emotions influencing mental health (Almadani & Alwesmi, 2023; Gross et al., 2019).

### Philosophical Paradigm

Our study uses qualitative tools and approaches to understand how people interpret and represent reality. We subscribe to Berger’s (1991) social constructivist view of reality, which recognizes that reality is context-dependent, influenced by society, culture, and language, and can be interpreted in various ways. Furthermore, our study subscribes to a community-based participatory research methodology, recognizing community members as co-researchers. Our study uses Photovoice to produce knowledge and reflect our broader methodological and theoretical perspectives.

### Study Design

Our study design was informed by Stephens and colleagues (2023) recent scoping review examining the use of Photovoice within mental health research involving adolescents to ensure rigor. The scoping review identified reporting issues relating to an absence of methodological information among Photovoice studies in adolescent mental health. These insights informed the design, collection, and reporting of data within this manuscript.

### Positionality Statement (MS)

I have multiple intersecting identities, some particularly relevant to this study, including being a White British adult, nurse, researcher, and female. The Social Identity Map, adapted from Jacobson and Mustafa (2019), details a review of my identities and can be found in Supplemental Material 1. The identities of NR involved in data coding are also presented in a Social Identity Map in Supplemental Material 2.

Firstly, as a White British adult, I have had access to education in Global North institutions, where I study at PhD level. My education has allowed me to access careers as a nurse and researcher, positions associated with respect and power. The assumptions individuals have toward these aspects of my identity could have affected power dynamics between the participants as co-researchers and myself and my choice of methods.

Secondly, I have previously lived in East London and nursed individuals residing there, including adolescents. This experience, alongside my female gender, may have shaped my interactions with adolescents and my understanding of the East London context. In turn, these factors may enhance my comprehension of local issues, cultural norms, and social dynamics affecting adolescents living in East London, potentially shaping my biases and the analysis of their data.

### Ethical Considerations

Our research protocol was informed by The Charter for Engaging Survivors (Perôt et al., 2018), a resource for planning research among individuals potentially affected by abuse. To protect participants’ confidentiality throughout this manuscript, we did not include participant-identifying information. Although adolescents aged 16 and over can legally consent for their involvement in UK research (Health Research Authority, 2024), parental consent was also obtained. The study was approved by the Queen Mary University of London Ethics Committee (approval number QMERC22.378).

### Public Engagement and Involvement Pre-Data Collection

Five adolescents co-designed our research protocol after responding to an email advert circulated by Future Leaders, an organization fostering leadership among East London adolescents.

Five adolescents were selected on a “first-come, first-serve basis” and shared their feedback on the study protocol during a 1-hour Zoom meeting on November 9, 2022. In exchange for their time and experience, adolescents were reimbursed with a £25 Amazon voucher and a certificate of participation. Furthermore, while these adolescents are acknowledged as co-authors, they did not engage in the research as participants to ensure their role remained independent of the research.

### Participant and Public Engagement and Involvement Post-Data Collection

On June 23, 2023, MS sent an email from her academic account, inviting adolescents who had participated in the study to an online Zoom meeting on July 6 to reflect on the themes produced by the wider research team. Two adolescents confirmed although they did not attend or provide a reason—however, another adolescent who had yet to respond to the original email attended. The five participants found our findings representative of their experiences, bolstering our findings’ validity, credibility, and confirmability.

Furthermore, a 2-hour online Zoom meeting on August 4 was organized for five adolescents undertaking work experience at our affiliated research unit to reflect upon the findings.

### Participant Recruitment

The study’s inclusion criteria were the following: they lived in East London, were between the ages of 16 and 19, had access to a mobile phone with a camera, were proficient in English, could attend all research meetings, and could provide written assent and parental consent. Our inclusion criteria were deliberately broad to promote inclusivity and gather diverse perspectives, promoting data sufficiency and aligning with our theoretical positioning.

We recruited adolescents enrolled or affiliated with Future Leaders to co-design our research program out of convenience because a senior researcher within MS’s affiliate research unit knew the organization’s leader. Future Leaders was the only organization among those contacted that responded. The organization’s ethos aligns well with the aims of the Photovoice method: empowering the excluded and reducing inequalities.

We used convenience and snowball sampling to recruit eight adolescents who were presently or had previously been enrolled in the Future Leaders program or were acquaintances of adolescents involved. Combining snowball sampling with convenience sampling facilitated data sufficiency by enabling our recruitment efforts to reach adolescents who may not have been accessible through convenient sampling alone.

Furthermore, our decision to recruit eight adolescents was influenced by previous Photovoice studies exploring adolescent mental health (Bashore et al., 2017; Dempsey, 2016; Georgievski et al., 2018; Liegghio, 2016). These studies indicate that data sufficiency may be achieved with a sample size of eight or fewer adolescents. The decision to include acquaintances rather than strangers recruited through other means was made to increase the quality of the data collected and make participants feel comfortable. Participants meeting the inclusion criteria were included on a “first-come, first-serve” basis. Participants were provided with a total of £75 for the time taking part in the study (this covered the time taken to take photos and take part in the focus group).

### Methodological Strategy

Our study employed Photovoice as the primary method for data collection. Photovoice is commonly used in adolescent mental health research to explore the social determinants of mental health by actively involving adolescents throughout the research process (Stephens et al., 2023). Thus, we selected Photovoice, believing it aligned with our research question and may help increase the participation of East London adolescents who remain underrepresented in mental health research. Additionally, Photovoice’s ability to provide detailed insights beyond those offered by traditional methods like interviews (Guell & Ogilvie, 2015) makes it relevant for exploring the complexities of mental health (Mans et al., 2021).

Photovoice research typically undergoes three key stages as outlined by its creators Wang and Burris (1997): (1) Participants receive training on how to use cameras, ethical considerations, and the power of photography. (2) Participants engage in group discussion to reflect upon the photographs they have collectively taken. (3) Lastly, participants analyze the data produced by their photos. These stages were operationalized in our study alongside other processes to fit our study’s needs, and they are described below.

### Research Stages

#### Collection of Demographic Data

MS collected demographic data, including age, gender, place of birth, and ethnicity, from the adolescents using a form adapted from the 2011 United Kingdom Census (Supplemental material 3). By collecting this data, we promote transparency, enabling readers to understand the findings within the context of the participant demographics. Furthermore, sociodemographic data collection is relevant, considering such factors are associated with mental health problems (Hubbard et al., 2021) and may be helpful for secondary analysis.

Proxy socioeconomic data related to the adolescents’ parental occupations and their subjective social status were collected by MS using the MacArthur Scale of Subjective Social Status (Adler et al., 2000). This scale asks participants to rate their perceived social hierarchy on a 10-rung ladder (Moss et al., 2023). It was chosen because its inherent subjectivity aligns with our theoretical position. Also, the scores individuals give themselves using the scale correlate with mental health outcomes (Rubin, 2020) and are relevant given our study’s focus on adolescent mental health. These data helped situate our findings and improve the reporting of Photovoice studies, which are limited within the field of adolescent mental health (Stephens et al., 2023).

#### Research Briefing and Ethics Discussion

Next, we invited adolescents to a 1-hour Zoom meeting on March 30, 2023, reminding them of the study’s purpose and aims (photographing things and places that make them feel happy or sad). During this meeting, we discussed the ethical considerations of conducting research and capturing photos in public spaces, emphasizing the importance of appreciating peoples’ privacy.

To ensure individuals’ protection and privacy, MS instructed adolescents to avoid taking photos of easily identifiable individuals, specifically in private areas. Instead, MS suggested that they photograph objects and places to represent people. These instructions followed the procedures detailed in the study protocol and mitigated the need for adolescents to consent to photograph individuals for research purposes. Moreover, MS explained that adolescents could take photos in public areas where photography is generally expected, although MS encouraged them to be discreet and avoid dangerous situations. Lastly, the adolescents were reminded that the photos were intended to serve as props for further conversation and that the quality and technicality of the photos were less important to the research.

Furthermore, we asked that adolescents not photograph sexual or explicit content, reminding them that we would destroy photographs containing such content, withdraw their participation, and escalate concerns as appropriate.

Notably, four adolescents did not attend this meeting, citing Wi-Fi issues, school, confusion over the meeting’s timing, and illness. We accommodated these adolescents by hosting separate online meetings later that day.

#### Data Collection: Part 1

From March 30 to April 12, 2023, adolescents collected 10 photos representing things and places that evoke their happiness or sadness. We instructed adolescents to send these images to MS’s institutional email. As a prompt to promote engagement, we sent a reminder email to the adolescents within a week of the initial training meeting.

#### Data Collection: Part 2

All eight adolescents attended a 1.5-hour in-person meeting on April 13, 2023. The venue for data collection was a private room within The Idea Store public library in Whitechapel, East London. We chose this location based on suggestions from adolescents who contributed to the research protocol’s design. MS facilitated the session, where adolescents were encouraged to caption their photographs and select one for further discussion by the group the following week. Adolescents had creative freedom when producing captions and selecting one photo for analysis and were not constrained by guidance that could have shaped their decisions. Only participating adolescents and MS were in the room throughout the research meeting, during which refreshments were available.

#### Data Collection: Part 3 and Data Analysis: Part 1

The following week, on April 20, 2023, the eight adolescents participated in a one-and-a-half-hour in-person focus group at the location and time of the previous meeting. We opted for a single focus group to prevent overburdening participants who were preparing for their exams and had also attended two research sessions. Also, a singular focus group ensured that the entire data set, incorporating other narrative and visual data, would be manageable for analysis by a small team.

Before the focus group, we asked adolescents to read and sign a “respect and kindness guidance” statement (Supplemental material 4), reiterating expectations concerning protecting their peers’ confidentiality, dignity, and safety. During the focus group, co-facilitated by MS and NR, adolescents discussed eight photographs collected and selected for further analysis by each of the eight adolescents. The adolescents used manifest and latent content analysis to examine the eight photographs, with the former exploring “the obvious” within photos and the latter exploring deeper meanings within the images.

The SHOWeD framework (Shaffer, 1985) supported the analytical discussion of each photograph, which was randomly selected and asked five questions: (1) What do you see here? (2) What is happening here? (3) How does this relate to our lives? (4) Why does this condition exist? (5) What can we do about it? MS and NR took turns asking the questions presented by the SHOWeD framework, with MS asking the majority. The questions were posed in an untimed, sequential order and adhered to the SHOWeD framework’s content. New questions were posed when the adolescents’ discussion of a particular question or associated prompt had dissipated. Mental health scholars often use the SHOWeD framework within Photovoice research involving adolescents (Stephens et al., 2023) and were thus not pilot-tested. Adolescents also had the opportunity to freely discuss the eight photographs at the end of the focus group.

The singular focus group discussion lasted 1.06 hours and was documented using two digital voice recorders. MS also collected field note observations that were triangulated alongside participants’ narratives of photos discussed during the focus group. This process enabled us to compare observations like laughter, response patterns, and vocal tone fluctuations with participants’ photo narratives. Our processes deepened our analysis and promoted data sufficiently by bolstering the credibility of our findings.

No individuals other than the participants, MS and NR, were in the room during the recording, although Participant 6 attended the meeting 35 minutes late. As a gesture of appreciation, refreshments were available, and each participant received a certificate of participation alongside a reimbursement of £90 (£75 for their involvement in the study and £15 to cover travel expenses). Providing financial compensation was approved by the Queen Mary University of London Ethics Committee. The rationale was that considering adolescents lead busy and active lives (Mawn et al., 2016) and are expected to attend multiple research meetings during their exams, the value of their time should be reflected and acknowledged. Furthermore, the value of the compensation aligned with the guidance published by the National Institute of Health Research (2024), mitigating the potential risk of coercing participation through financial incentives.

#### Part 2: Data Analysis

Data analysis was an iterative data-driven process that began in May and ended in February 2023.

Firstly, the focus group transcript was analyzed using Braun and Clarke’s (2021) reflexive thematic analysis alongside captions followed by photographs, a preferential decision. Data analysis stopped when we produced rich, informative insights which answered the research question and represented adolescents’ diverse views and experiences.

Reflexive thematic analysis is a flexible method that belongs to a qualitative paradigm and appreciates the researcher’s active and subjective role in producing knowledge (Braun & Clarke, 2021). Reflexive thematic analysis is coherent with our theoretical research approach, and its inherent flexibility was beneficial to analyzing various data formats collected in our study. Our realization of the reflexive thematic analysis is detailed below.

##### Phase 1: Familiarization

MS and NR acquainted us with the data in various ways. We independently immersed ourselves in the data by repeatedly listening to the focus group’s audio recording, reading the transcript and captions, and viewing the images. Data familiarization occurred alongside other stages of reflexive thematic analysis, allowing us to remain “close” to the data as the analysis progressed.

##### Phase 2: Coding

MS and NR independently annotated printed and electronic versions of the transcript, captions, and photographs with codes. We applied two codes to data segments: semantic codes, which attribute apparent meaning, and latent codes, which attribute deeper meaning.

Firstly, we used semantic codes to identify “the obvious” elements within the entire data set. This process facilitated our understanding and ability to describe the data and provided a foundation for deeper and more interpretive analysis.

Next, we complemented our semantic coding by applying latent codes to the transcripts and captions. Applying latent codes allowed us to ascribe more profound meaning and interpretation to the written data, which would have unlikely been captured by semantic coding alone. Through latent coding, we could recognize nuances and contradictions within the data, enhancing the thick description of our data analysis.

We did not apply latent codes to the visual data because we anticipated difficulties assigning hidden meanings or implicit messages to non-textual data. Moreover, compared to the narrative data, applying latent codes to the visual data could lead to greater misinterpretation of visual content due to our unfamiliarity with the context of the photos and a lack of understanding surrounding the decision-making process by the adolescents who took them.

The coding process occurred in diverse settings, including outdoor environments such as gardens and beaches and indoor spaces like the author’s bedroom and kitchen, to promote diverse perspectives. Geographically, coding occurred across various locations, including London, Cornwall, Edinburgh, and Brazil, and at different times of the day. MS and NR also met regularly to discuss how their identities impacted the coding process.

Finally, MS and NR discussed and compared our independently organized code labels and corresponding data segments, reconciling any differences to co-produce agreed-upon codes with their corresponding data segments in an Excel spreadsheet. This process of double coding, followed by discussions, promoted credibility, validity, and confirmability while enabling data handling and management and the development of preliminary themes.

##### Phase 3: Generating Provisional Themes

Next, MS and BR produced provisional themes by recognizing patterns of shared meaning across the code labels, which were structured around relevant keywords such as animals, entertainment, and violence.

##### Phase 4: Forming and Reviewing Themes

MS and BR developed themes over many iterations by restructuring provisional themes and associated data around a central idea. In doing so, we created themes of analytical profundity, complexity, richness, and relative importance to the research question. We removed candidate themes that were “thin” or lacked a central idea and transferred data segments to other themes. Provisional themes, including the candidate themes that comprised them, were reviewed by the wider research team and developed further following feedback.

##### Phase 5: Refining, Defining, and Naming Themes

MS named each theme by writing a short descriptive paragraph defining the central meanings and analytical direction illustrated within each theme. Following consultation with the broader research team and during the write-up of this manuscript, these descriptions were refined and developed into short, impactful theme names.

##### Phase 6: Writing Up

MS carried out the writing and editing of this manuscript in collaboration with the broader research team, including some adolescents who participated in the study. Several manuscript versions were produced during the write-up, strengthening the analysis and presentation of insights.

## Findings and Discussion

The adolescents collected 81 photographs printed onto 4 × 6-inch photographic paper at an East London printing shop. The 81 photos represent raw data collected and curated by the adolescents. They are visual testimonies that reflect and frame their social realities concerning their happiness and sadness.

Table 1 presents the characteristics of the adolescents co-producing our research. Our sample comprises predominantly female adolescents of varying ages, aligning with previous Photovoice studies examining adolescent mental health (Stephens et al., 2023). This tendency could be attributable to the feminist theory guiding Photovoice (Wang & Burris, 1994), potentially leading to higher participation among females compared to males. Furthermore, our study primarily represents participants born within the United Kingdom who belong to an ethnic minority group. Our findings echo those elicited from the 2011 Census (Office for National Statistics, n.d.). They may also reflect the sample of adolescents from Future Leaders, which empowers adolescents from underrepresented backgrounds (Future Leaders, 2024).

**Table 1.** Participant Characteristics.

Participant-described gender
Female, *n* = 7
Male, *n*= 1
Age
16, *n* = 2
17, *n* = 5
18, *n* = 1
Participant-described place of birth
United Kingdom, *n* = 2
England, *n* = 5
Italy, *n* = 1
Participant-described ethnicity
Sri Lankan, *n* = 1
White British, *n* = 1
Bangladeshi, *n* = 2
Pakistani, *n* = 3
Other mixed or multiple ethnic backgrounds: half
Black African and Half Arab, *n* = 1

Furthermore, most adolescents reported that their parents/guardians held service-related jobs, such as portering, chefing, beautician, salesperson, shop assistant, health care assistant, single mum, butcher, and teacher assistant. Our finding may have influenced adolescents’ mean average score of 5 on the MacArthur Subjective Scale of Perceived Social Status, suggesting our sample adolescents perceive their social status modestly. These results are potentially significant for our research utilizing Photovoice, a method known to empower individuals (Macdonald et al., 2022). Therefore, Photovoice may increase participation among adolescents lacking high self-perceived social status.

The following section of our manuscript will present and discuss four major prevailing themes relating to nature and the built environment, the intersection of which is captured in our manuscript title. Our four themes are named: (1) The existence of nature is embraced, but its precarious existence is daunting. (2) Nature provides a limited respite from psychological stress, boredom, and artificial environments. (3) The urban environment is imposing, inspiring, and a symbol of access. (4) Visually appealing areas are paradisical and are too good for East London. Each theme incorporates elements of other central organizing concepts, including global climate crisis, religion, and education, demonstrating the interplay of social determinants of adolescent mental health across micro, meso, and macro levels.

### The Existence of Nature Is Embraced, but Its Precarious Existence Is Daunting


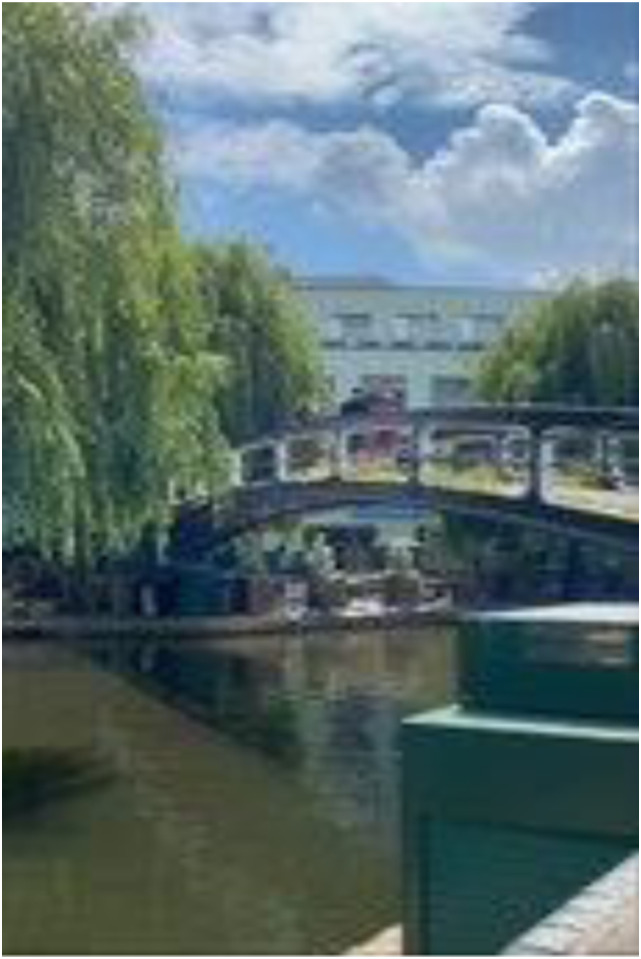


Photo 1, Participant 3

Adolescents in East London are grateful for their interaction with nature within London. Participant 1 evidences this claim in their caption: “There are always places around to help us appreciate the nature around us,” which may be echoed in Participant 3’s photo (left). Similarly, Participant 2 expresses how opportunities to appreciate nature occur at particular times of the day while captioning a photo containing a sunset. They write, “Admiring the sunset as the day is finishing.” Considering the data, adolescents seem to highlight the prevalence of nature across London, which is evident in adolescents’ bountiful collections of photos featuring trees, rivers, sunsets, and flowers. Our data supports previous evidence, suggesting adolescents appreciate nature (Kaplan & Kaplan, 2002). Thus, our findings may be relevant in understanding determinants influencing adolescents’ happiness and sadness, given the link between nature’s abundance and happiness (Houlden, 2021).


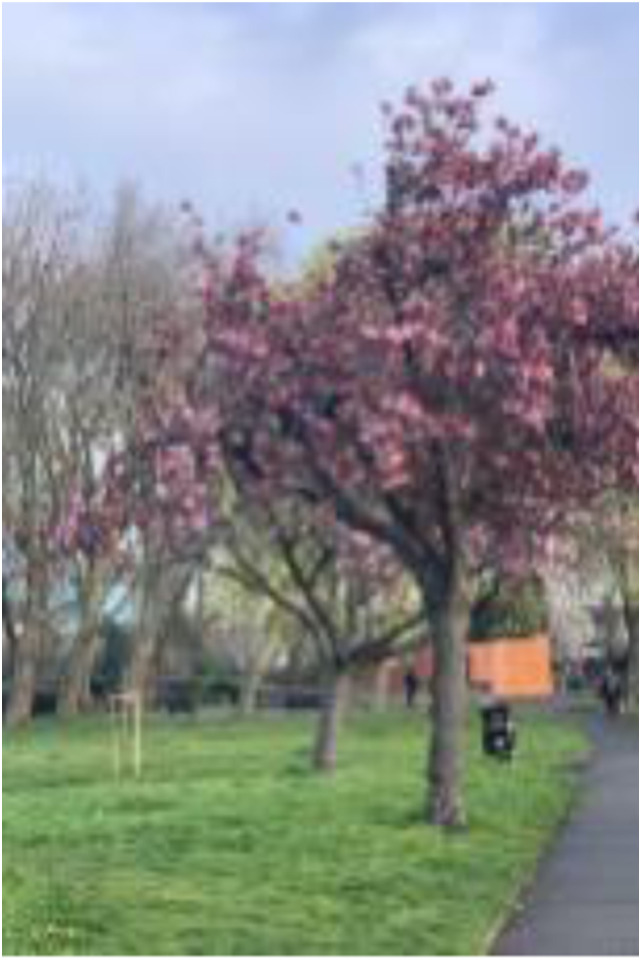


Photo 2, Participant 2

Furthermore, our finding may also be relevant to the London context, which the Greater London Authority claims as one of the greenest cities (n.d.). However, our findings may have been influenced by the timing of our study, which coincided with spring. During this time, adolescents felt “happy” (Participant 5) and were “bombarded by flowers” (Participant 1), including “blooming blossoms” (Participant 5)*,* as illustrated in Photograph 2 (left). Thus, the occurrence of spring may have shaped adolescents’ decisions to collect predominantly outdoor photos featuring nature.


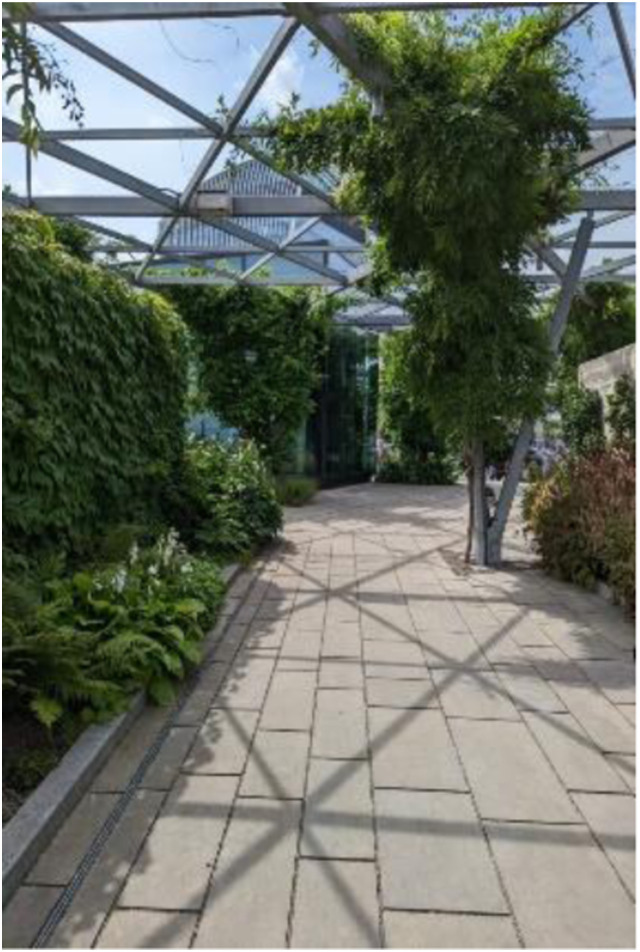


Photo 3, Participant 1

Strikingly, Participant 6 perceived that eco-concerns politically determine the existence of nature within London. This was evident in their comments on a photo containing trees in front of buildings (photo 8, below). They remark, “Everyone is taking in more like the climate crisis … Like the government and people are … like councils are trying to show more naturalistic features around the busy like buildings.” This sentiment may be represented in photo 3 (left) and collaborates with research reporting how environmental concerns influence the number of green spaces (Bush, 2020; Wilkerson et al., 2018). In their recent study of 107 participants, Stieger and colleagues (2022) report how exposure to nature influenced happiness. Thus, our findings could suggest that political decisions related to the provision of nature in London may influence adolescents’ happiness and sadness.


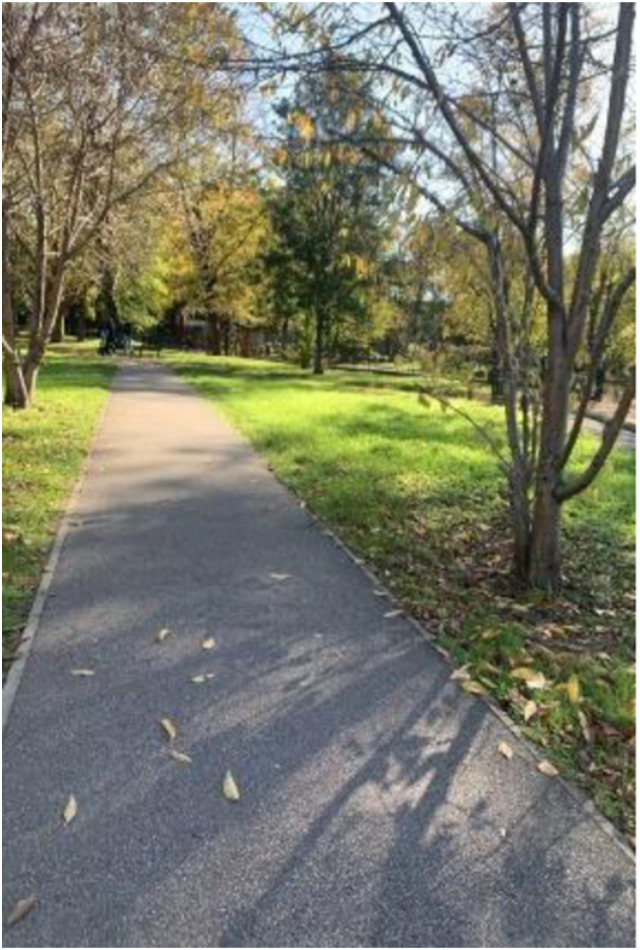


Photo 4, Participant 2

Moreover, adolescents residing in East London are connected and near nature. This is evident in Participant 2’s caption of photo 4 (left): “taking a walk in the park on my way to school.” Similarly, Participant 4 explains how the River Thames is “close to where I live,” while Participant 3 underscores their proximity to public green space in their caption of a photo containing a park. They write, “… This is only a few minutes from my house. I don’t have a particular reason why I like it. I think it’s because I’ve made so many memories there over the last 15 years.” These quotes may suggest that adolescents’ proximity to nature connects them to their histories, as echoed in Participant 2’s caption of a photo featuring a body of water (photo 6, below): “The nostalgic duck pond near my old secondary school.” Our findings connect with evidence suggesting that proximity and connection to nature determine happiness (Balseviciene et al., 2014; Capaldi et al., 2014).

However, our findings should be considered alongside our sample, which is predominantly female. According to a recent cross-cultural study (Rosa et al., 2023), females connect with nature more strongly than males, which may explain our findings. Nevertheless, our finding may be beneficial in understanding social determinants of adolescents’ happiness and sadness and their underlying mechanisms.


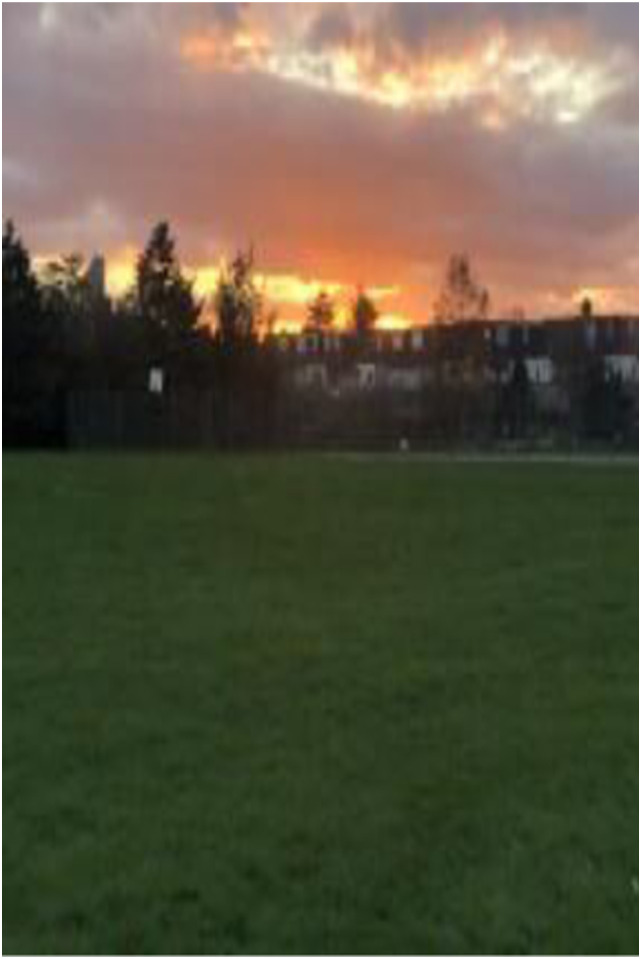


Photo 5, Participant 1

Notably, adolescents acknowledge how exposure to nature promotes appreciation and respect toward nature within themselves and others. This claim is evident in Participant 5’s analysis of a photo containing trees among a cluster of buildings (photo 8, below). They suggest, “We should appreciate the nature around us … and avoid littering.” Participant 3 echoes similar sentiments while commenting on a photo containing public green space (photo 5, left). They remark, “I think it should be taken as a reminder to appreciate nature that surrounds us.” Similarly, while discussing a photo containing a pond (photo 6, below), Participant 7 highlights how the person taking the photo “may have come to feed the ducks … and then they admire how beautiful the water actually looks.” These statements appear to uphold findings from a nationally representative survey by Alcock and colleagues (2020), reporting how nature promotes appreciation among adolescents.

Furthermore, our findings may be relevant in understanding determinants of adolescents’ happiness and sadness since appreciation of nature is correlated with reduced sadness and increased happiness (Craig and colleagues, 2015). However, it is important to contextualize our findings in the aftermath of COVID-19. During the pandemic, visits to green space were reduced (Burnett et al., 2021), and appreciation for nature grew (Lemmey, 2020), possibly influencing our findings.


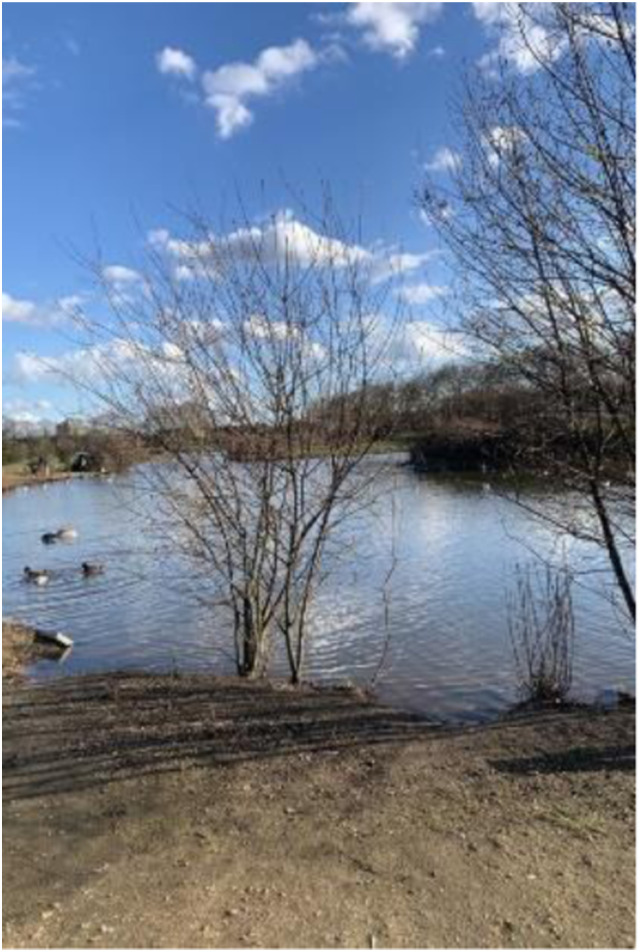


Photo 6, Participant 2

Moreover, adolescents recognize that natural spaces offer opportunities to enhance their happiness. For instance, Participant 5 highlights how natural spaces provide opportunities for “people to relax and play sports.” This notion was evident in Participant 3’s comments while analyzing a photo featuring a walkway and a pond (photo 6, left). They comment how areas containing waterbodies “exists so all people from, like ranging ages of the community can hang out. But like specifically teenagers, it is a free thing to do in London … So it is a way to have fun.”

Similarly, when referring to a photo featuring a field, Participant 3 explains how they “offer so many opportunities, like during the summer for teenagers like picnics or just hang out sessions.” These quotes appear to suggest that natural spaces provide “easily accessible” (Participant 8) and affordable opportunities for leisure and social interaction, which influence happiness, according to an international survey by Wang and Wong (2014). Consequently, our findings may help us understand how the opportunities provided by nature potentially determine adolescents’ happiness and sadness. Our findings could also be relevant given that adolescents tend to lack money (Wang et al., 1996) and the current UK cost of living crisis (Singh & Uthayakumar-Cumarasamy, 2022).

While adolescents recognize the abundance and benefits of nature within London, Participant 1 repeatedly highlights how “you have to look for nature.” This sentiment was reinforced by their comments on a photo featuring a pond with ducks (photo 6, above). They remark, “Encourage people to just look at the beauty around them … because it’s not always going to be around you.” Elaborating on this, Participant 1 attributes the fragility of nature to global environmental challenges while captioning a photo of a blossoming tree. They write, “As deforestation becomes more common, we don’t see nature as much as we should.” These quotes possibly suggest that the aesthetic benefits derived from nature are transient despite its perceived abundance and should be embraced. Our finding quote may be salient in the context of the global climate crisis, which adversely impacts individuals’ happiness according to a recent scoping review by Charlson and colleagues (2021). Thus, our findings may indicate how broader environmental issues can influence adolescents’ appraisal of nature, potentially affecting their happiness and sadness.


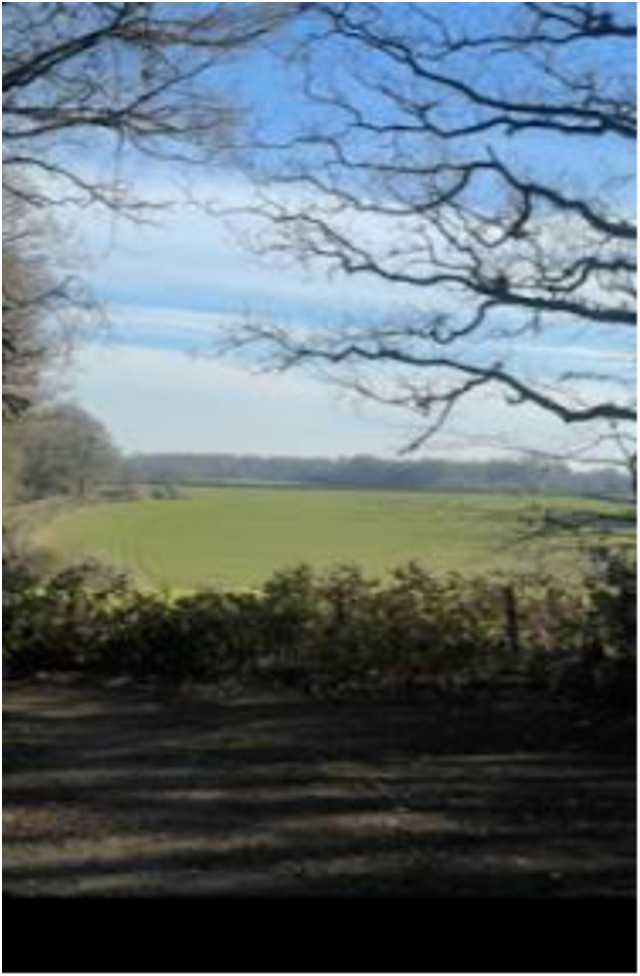


Photo 7, Participant 6

Adolescents call for “a balance” (Participant 2) between maintaining London’s natural spaces and urban development. This sentiment was evident in Participant 5’s comments when analyzing photo 8 (below), featuring trees among lofty buildings. They remark, “I think instead of building more buildings, they should plant more trees and encourage nature.” Similarly, Participant 3 suggests that “we need to encourage green spaces from being built on.” These quotes give the impression that adolescents’ affinity for public green spaces, as represented in Participant 6’s photo (photo 7, left), could conflict with “the regeneration and gentrification happening in London right now” (Participant 3). Research by Siddique and Uddin (2022) examining green space distribution in an Asian city found that green spaces diminish alongside urban expansion; our findings may apply to East London, which has undergone urban development and lacks green spaces (Department of Levelling Up, Housing and Community, 2022; Zylva de et al., 2020) and likely affects adolescents’ happiness and sadness.

### Nature Provides a Limited Respite From Psychological Stress, Boredom, and the Artificial


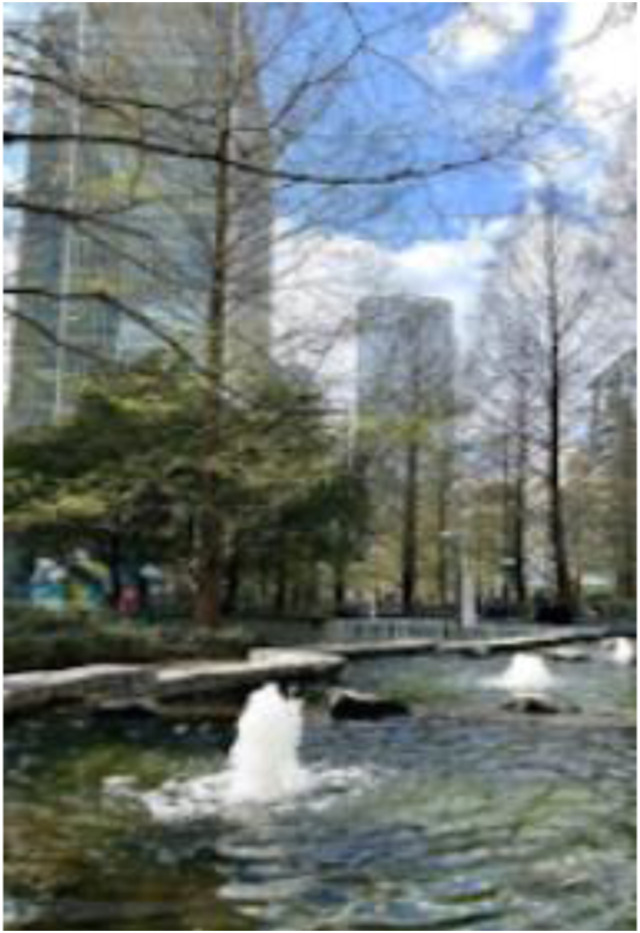


Photo 8, Participant 4

Adolescents living in East London understand how nature soothes psychological stress. Participant 8 provides evidence of this in their reflection of a photo containing water and trees (photo 8, left). They recognize the setting as “somewhere someone can go for some quiet … if stressed or have something on their mind.” Participant 4 reinforces the therapeutic benefits of nature in soothing stress while commenting on the same photo. They describe the setting as “a place where they can go whenever they feel stressed.”

Similarly, participant 5 expresses the solace they find in sunsets while analyzing a photo containing a sunset (photo 5, above). They comment, “When I am overwhelmed with stress, especially during A-levels, I always look outside the window and see the sunset, and it helps me calm down and not think about exams.” Our findings appear to reinforce Collado and colleagues’ (2017) literature review, which recognizes how nature promotes coping and resilience toward stress. They also appear to resonate with Ulrich’s seminal (1983) Stress Recovery Theory, positing that nature buffers against psychological distress, potentially affecting adolescent’s happiness and sadness.

However, readers should consider the timing of our study, which was conducted while adolescents were preparing for A-level exams. During this period, adolescents reported being “really busy and stressed” (Participant 2) and may have been more attuned to the therapeutic benefits of nature in reducing any feelings of sadness.


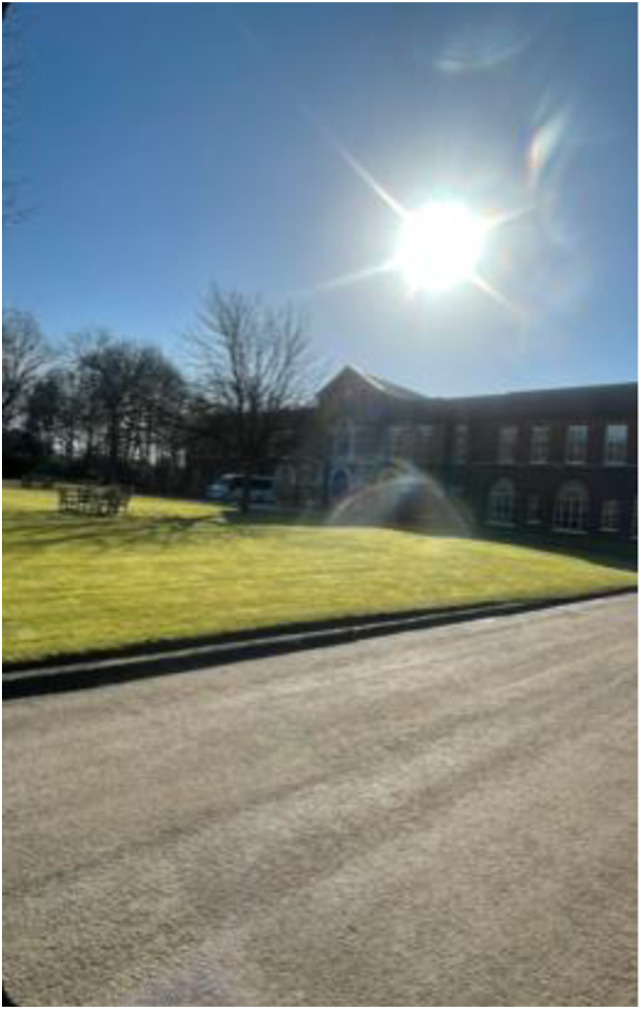


Photo 9, Participant 6

Furthermore, adolescents recognize that nature exists to “just be beautiful” (Participant 2), influencing their emotions and providing a sanctuary from a humdrum life. For instance, in Participant 6’s caption of a photo featuring a blue and sunny sky (photo 9, left), they write, “This is a picture of the beautiful sun. It makes me happy as I feel more fresh and energised.” Likewise, Participant 7 echoed a similar sentiment while analyzing a photo of a sunset (photo 5, above). They remark, “From my perspective, I feel like what is happening is that someone just wants to escape their mundane life, and they need to see something more beautiful, so they watched the sunset, which is quite stunning.” These quotes seem to echo Kellert and Wilson’s (1993) biophilia hypothesis, hypothesizing that individuals are instinctually attracted to beautiful nature, which Igou and van Tilburg (2019) more recently linked to reduced boredom. Thus, our finding may hold relevance in understanding determinants influencing adolescents’ happiness and sadness, considering boredom promotes individuals’ pursuit of happiness (Bench & Lench, 2013). However, readers should be aware that our study coincided with Ramadan, possibly increasing adolescents’ observation of natural processes such as sunrises and sunsets, which are frequent in our data.

Simultaneously, adolescents recognize the mediating role of nature within the artificial environment. This suggestion is evident in Participant 3’s comments about a photograph showing trees and a water feature amid tall buildings (photo 8, above). They claim, “It is in a pretty urbanised area, so it is probably to break up the buildings.” Participant 7 echoes a similar sentiment regarding the same photo. They state, “… it allows us to take a step back from all the buildings … Due to industrialisation and just take in nature.”

Similarly, while analyzing the same photo, Participant 8 highlights how “in so many buildings it’s a place to take a break and just enjoy the natural environment, which is really important to our daily lives.” These quotes may indicate that nature provides adolescents with a visual escape, possibly by altering the aesthetic of dense urban environments, which Skrede and Andersen (2022) link to adolescent sadness. Thus, our finding may be important in understanding determinants of adolescents’ happiness and sadness and appears to synchronize with previous research correlating nature with reduced stress (Asgarzadeh et al., 2012).


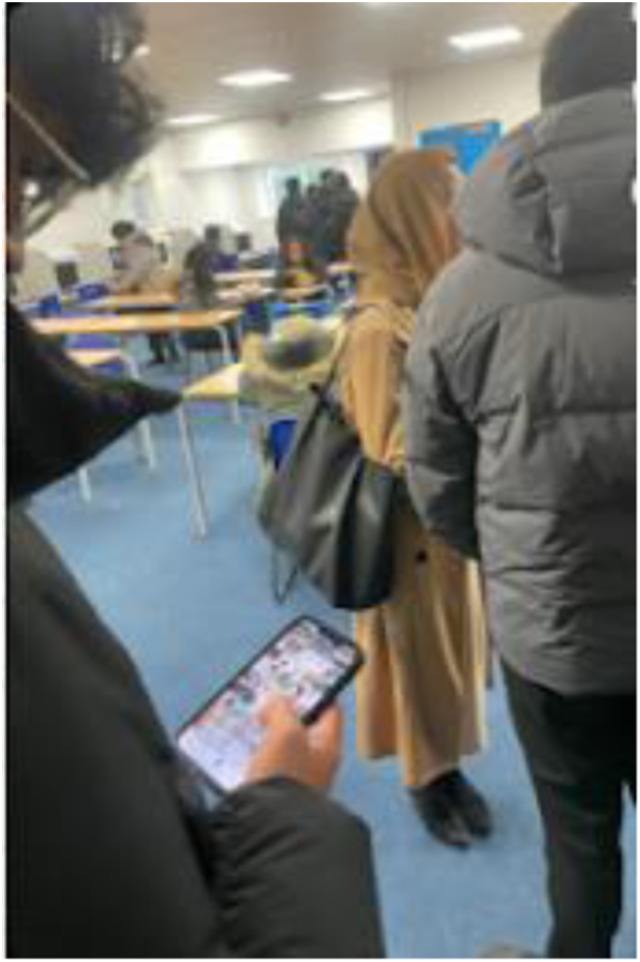


Photo 10, Participant 6

Notably, Participant 8 recognizes how nature reallocates their attention away from their mobile phone, captioning a photo of a walkway alongside the River Thames (photo 14, below). They explain, “I enjoy walking here with a friend that lives here too. It is a great chance to get off our phones and spend time together in real life.” This comment could suggest that nature reduces adolescent mobile phone use, which a 2-year study by Minor et al. (2023) associates with sadness. Also, our findings could be relevant given the prevalence of smartphone addiction in the United Kingdom (Sohn et al., 2021), which may be represented by Participant 6’s photo (left). In their mixed-methods study, Nielsen and Arvidsen (2021) report a link between mobile phone use and reduced adolescents’ time spent outdoors. Consequently, our finding may be compelling in understanding determinants influencing adolescents’ happiness and sadness and how they intersect and offset each other.


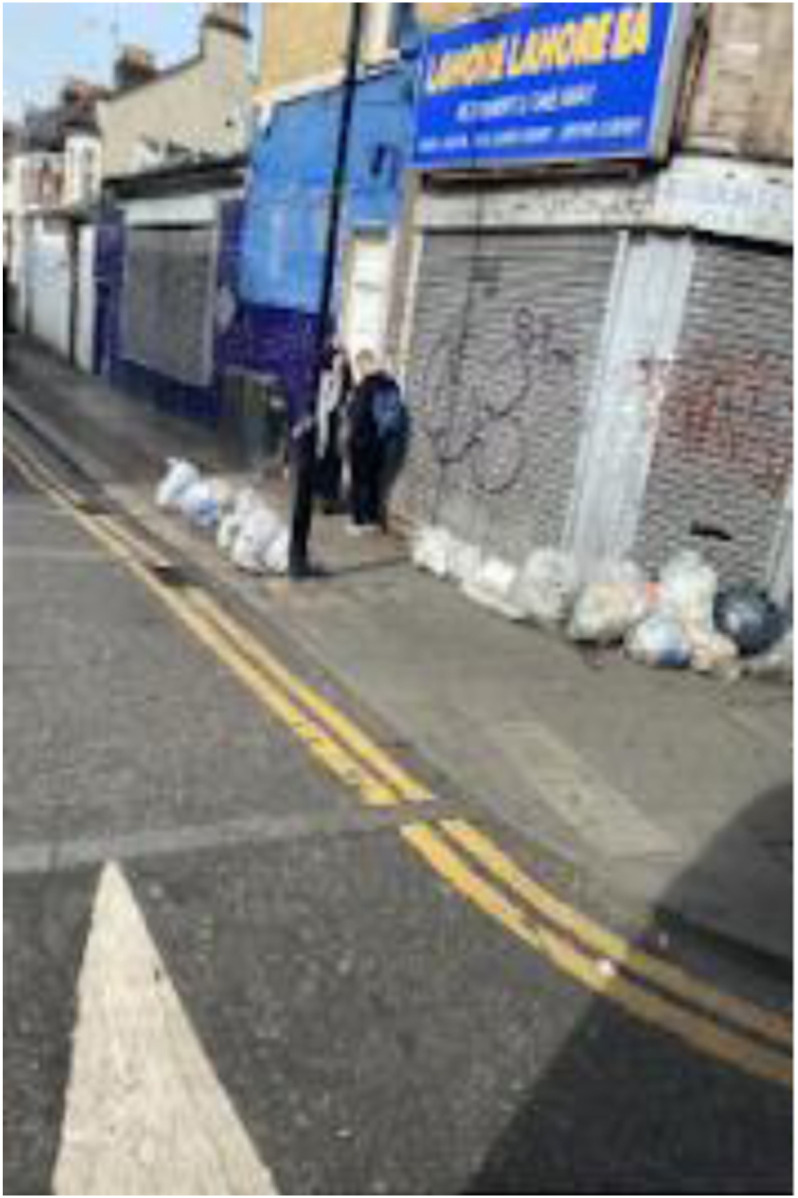


Photo 11, Participant 5

However, adolescents recognize that the escapism offered by natural spaces is limited. This idea was alluded to in Participant 7’s caption of their photo featuring green space close to residential housing. They write, “Escaping from the world at the park … only to see the worst of the world.” This quote could infer that exposure to adverse environmental conditions blights the escape sought by adolescents and offered by natural environments. In their analysis of secondary sources, Srivastava (2009) claims that stressors, including pollution, are higher in urban environments. This claim resonates in Participant 5’s repeat references to “dirty rubbish in the road.” For instance, while captioning a photo containing litter (photo 11, left), they write, “Litter damaging the city. Seeing the litter on the streets can be very upsetting and damaging to the environment, sometimes even blocking the roads.” These findings seem to convey that adolescents are reactive to urban stressors, which increase their sadness, possibly offsetting the happiness they could derive from natural spaces. Thus, our findings may be important in understanding urban-specific determinants influencing adolescents’ happiness and sadness.

Nevertheless, adolescents highlight how incorporating nature into urban design should be “the standard for all areas” (Participant 3). This idea was evident in Participant 7’s comments about a photo of trees and skyscrapers behind a water fountain (photo 8, above). They suggest the photo should be used:

as an example of what areas we should have in more like localised places and stuff. I think it should be used as an example to create more spaces like this for people to get out from the world and just like go to their little world.

These quotes seem to insulate that adolescents associate urban design incorporating nature with comfort and connection. Our finding may have utility in understanding determinants of adolescents’ happiness and sadness, considering the relationship between naturalistic urban design and happiness (Awadhesh Kumar, 2017). Moreover, our findings are likely pertinent to adolescents often excluded from urban planning processes (Nordström & Wales, 2019) and potential opportunities to “design in” their happiness.

### The Urban Environment Is Imposing, Inspiring, and a Symbol of Access


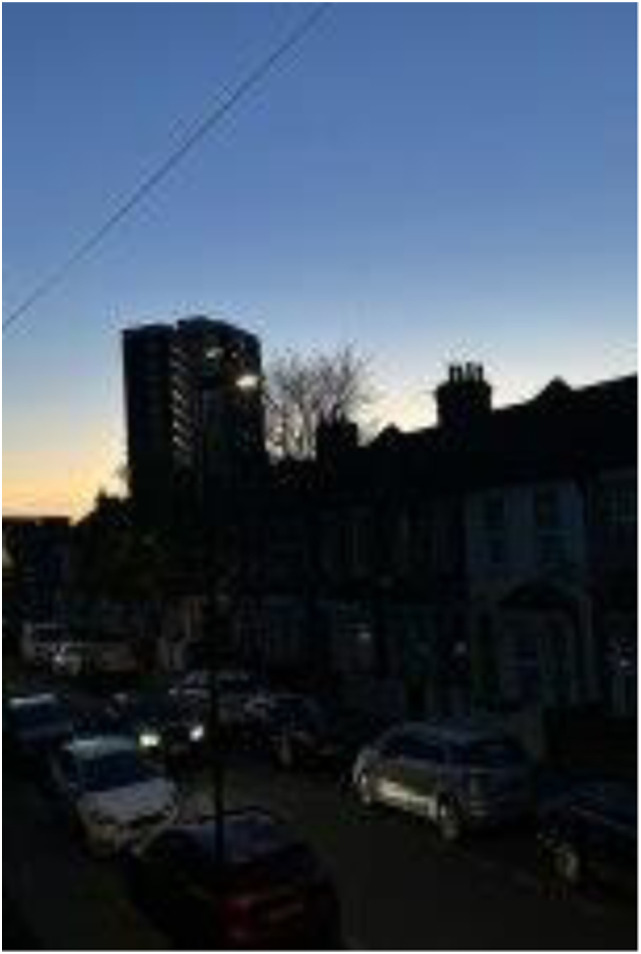


Photo 12, Participant 4

Adolescents allude to how London’s compact and towering built environment imposes upon them and lurks “in the background behind trees.” This comment is supported by Participant 4’s photo (photo 12, left) and other photos in this manuscript. Additionally*,* while reflecting on a nighttime skyline photo (photo 14, below), Participant 8 remarks how “there are places like this to see all over London.” These quotes appear to suggest that an unbroken 360-degree view of buildings surrounds East London adolescents. Adolescents reflecting on this data remarked how buildings make them feel like caged animals. These reflections seem to connect with questionnaire research by Baiz and Hoskara (2020), attributing high-rise environments to a rat-cage mentality. Our findings may also support research linking density-built environments and mental health problems (Knöll et al., 2017) and may be important in examining social determinants affecting adolescent happiness and sadness.


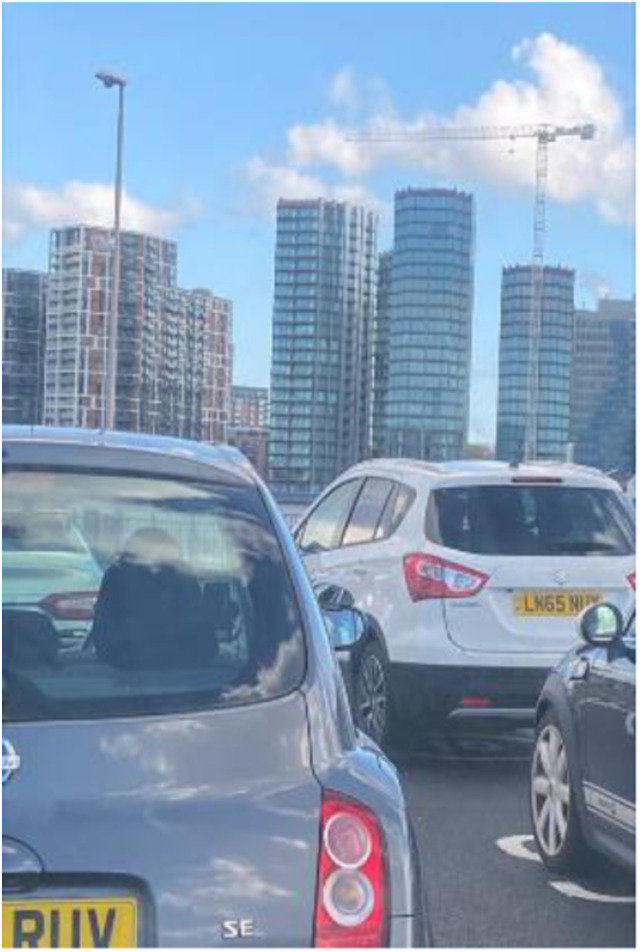


Photo 13, Participant 5

However, adolescents are simultaneously “starstruck” (Participant 1) by towering buildings in London. This idea resonates in Participant 5’s caption of their photo containing tall buildings (photo 13, left). They write, “I admire tall buildings. Seeing these tall buildings gave me inspiration and ambition for the future as I wish to one day work in one of them.” This quote could suggest that adolescents derive hope from high-rise buildings, reinforcing how structures symbolize hope and progress (Hardyman, 2016). Therefore, our finding may be important in understanding the determinants of adolescent happiness and sadness, particularly as awe buffers against hopelessness (Tarani, 2017).


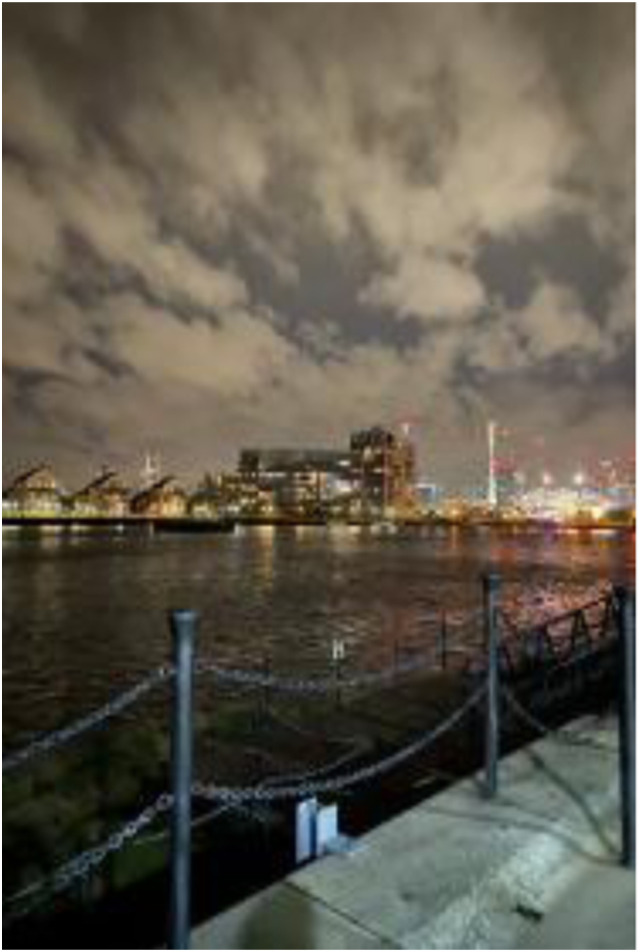


Photo 14, Participant 8

However, adolescents also highlight that living near tall buildings could be irritating, which could offset the awe and potential happiness elicited from such structures. This reflection may explain why adolescents across the data predominately refer to “the view” rather than specific buildings. For instance, while commenting on a photo containing the London skyline (photo 14, left), Participant 7 remarks: “I’ve gone with my friends to like, go and see the view and stuff and I think that it’s just a great way to escape.” This quote could indicate that observing urban buildings from a distance provides therapeutic benefits and opportunities for adolescents to socialize. Our findings seem to reinforce previous research by Yaran (2016) associating view corridors with positively perceived building aesthetics, which may affect adolescents’ happiness and sadness.


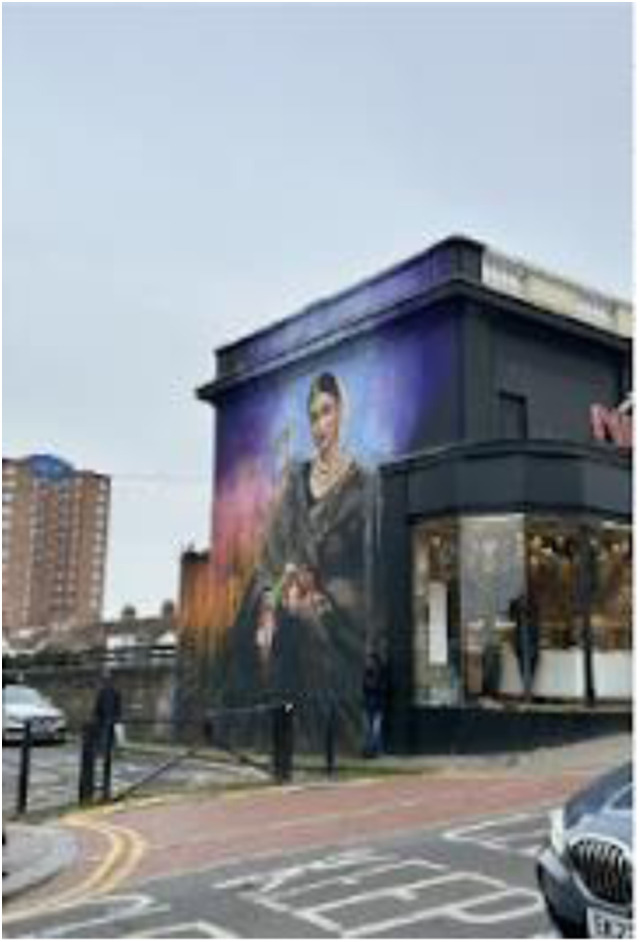


Photo 15, Participant 4

Additionally, adolescents observe how specific building features, such as artwork, can evoke positive emotions when interacting closely with buildings. This idea was evident in Participant 4’s caption of their photograph (photo 15, left): “Art is everywhere—happy.” Furthermore, while commenting on a photo containing a temple (photo 17, below), Participant 7 remarks:

It’s so interesting about whoever, like, made it, how they will manage to fit such a huge building in such a small part of like an area and stuff, and how like, like they’ve used such like detailed designs that make it stand out when you walk past it.

These quotes appear to convey adolescents’ mindfulness and reactivity to building aesthetics, which are known to influence well-being (Kent et al., 2017) and likely affect adolescents’ happiness and sadness.

Furthermore, adolescents recognize how “the view” of London symbolizes proximity to the city center while commenting on a photo featuring the London skyline (photo 14, above). Participant 3 comments:

It shows that even though this might only be 20 minutes from an area that I live in, like in East London, this looks like it’s closer to central, that we’re still very close to the nightlife and being able to enter the city and be a part of it.

Likewise, while reflecting on the same photo, Participant 2 highlights how “it relates to my life because it’s like an area where we could easily go because we live in East London.” These remarks seem to suggest that adolescents recognize the convenience of living in East London due to its accessibility to opportunities available within Central London. Our findings reflect those from a cross-cultural study by Mouratidis and Yiannakou (2022) and could indicate that East London adolescents’ proximity to the city center plays a role in determining their happiness and sadness.


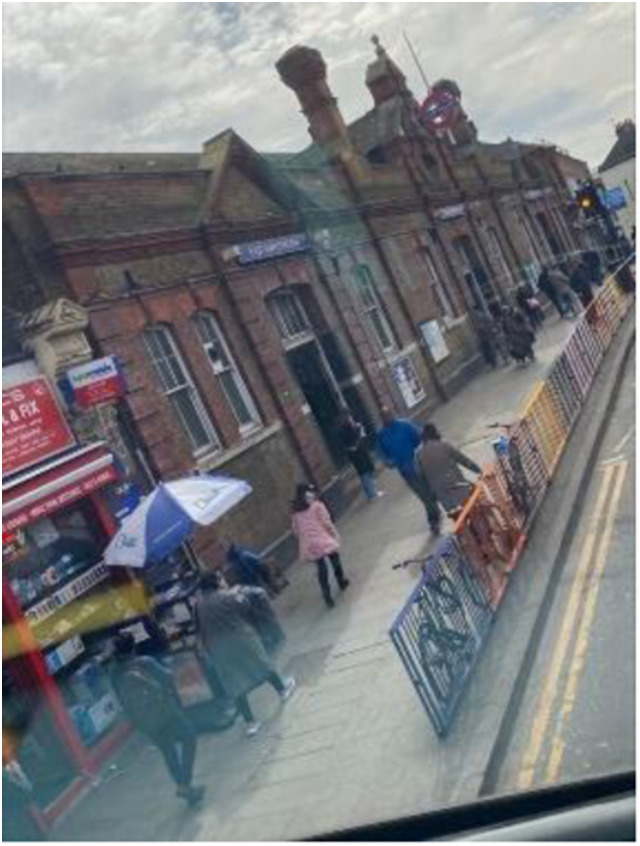


Photo 16, Participant 5

However, our finding may be attributable to the recent introduction of the Elizabeth Line, which provides fast train connections between East London and Central London (Transport for London, 2022). For instance, in their caption containing a train, Participant 3 writes, “This is a shot of the Elizabeth Line at Chadwell Health Station. I took this because I feel so grateful to have access to Central London within 30 minutes.” This quote infers that adolescents place importance on London’s transport network, supported in Participant 5’s caption of a photo featuring a tube station (photo 16, left). They captioned the photo, “Travelling around the world,” explaining that “using the tube is vital for me as it allows me to gain access to multiple areas that I can discover and also travel to school.”

These statements seem to support López-Ruiz and colleagues’ (2021) research, which found a positive correlation between transport services and quality of life among 933 individuals. However, this link may not translate for adolescents using buses in East London, which Participant 7 claims “aren’t for the weak.” Our findings could imply that despite the significance adolescents place on London’s transport system, disparities exist in their appraisal of various modes of public transport. These differing evaluations of public transport may be beneficial in understanding how public services determine adolescents’ happiness and sadness.

### Beautiful Areas Are Paradisical and Are Too Good for East London


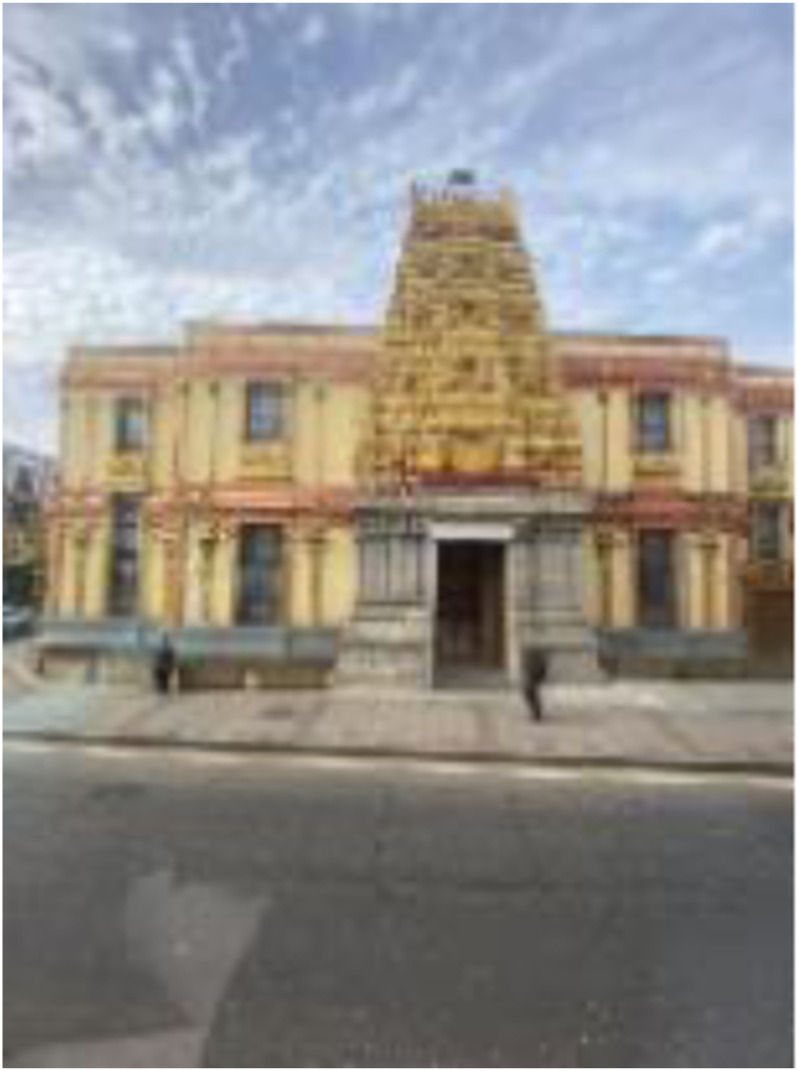


Photo 17, Participant 5

Adolescents perceive “beautiful” urban environments as otherworldly and idyllic. For instance, Participant 5 describes a photo containing a temple (photo 17, left) as a “place of Paradise” in their caption. Similarly, Participant 7 considers a photograph of trees among buildings (photo 8, above) heavenly. They remark:

It seems like a utopia of some sort, and it relates to my life because I study Utopia in school … if I think about an image of what looks like a perfect world, this would be my perfect world.

Our finding appears to chime with an ethnographic study by Roe and Aspinall (2011), who recognize how adolescents’ fondness for areas is socially and physically influenced. Consequently, our findings could indicate that adolescents’ academic exposure can alter perceptions of beauty within their environment, potentially influencing their happiness and sadness.

Moreover, adolescents recognize that tranquil spaces across London provide an escape from daily life stresses. This sentiment was evident in Participant 8’s analysis of a photo containing trees against a backdrop of buildings (photo 8, above). They remark, “It kinda looks like an escape area, so like just somewhere where someone can go and like some quiet. You know if like stressed or have something on their mind, just like a place to escape.” Equivalently, Participant 4 refers to a photograph containing a temple (photo 17, above) as “a place that you can go to or like visit when you feel overwhelmed and stressed.” These remarks support Participant 1’s claim that the urban environment “can be peaceful” while contradicting their description of London as “chaotic and busy.” These opposing perspectives could be explained by how tranquil environments enable individuals to become aware of the previously unnoticeable (Wong, 2022). Furthermore, our findings seem to align with Herranz-Pascual and colleagues’ (2019) observational study describing how serene environments, a predictor of happiness (de Vries et al., 2021), increase emotional restoration. Thus, calm areas could offer therapeutic benefits which may alter adolescents’ perception of their environment, potentially influencing their happiness and sadness.

Notably, adolescents recognize that visually appealing urban environments exist “because the councils want nicer places to look at” (Participant 7) and are “trying to show more naturalistic features” (Participant 6). Participant 1 substantiates these claims while analyzing a photo containing a water foundation among tall buildings (photo 8, above). They reflect:

I think because … just like the term littering is much more popular now. So the Council, the government, wants to do more about it because it will affect us in the future. So they make places like these to make people more mindful of what they do.

This quote seems to suggest that environmental concerns can promote political action, which could influence behavior. Our finding appears to mirror Gobster and colleagues’ (2007) essay describing how maintained environments promote pro-environmental behavior, a determinant of happiness (Kasser, 2017). Thus, our findings may indicate how socio-political action (or inaction) influences adolescents’ happiness and sadness and may be relevant considering adolescents spearhead environmental activism (Pickard, 2019).


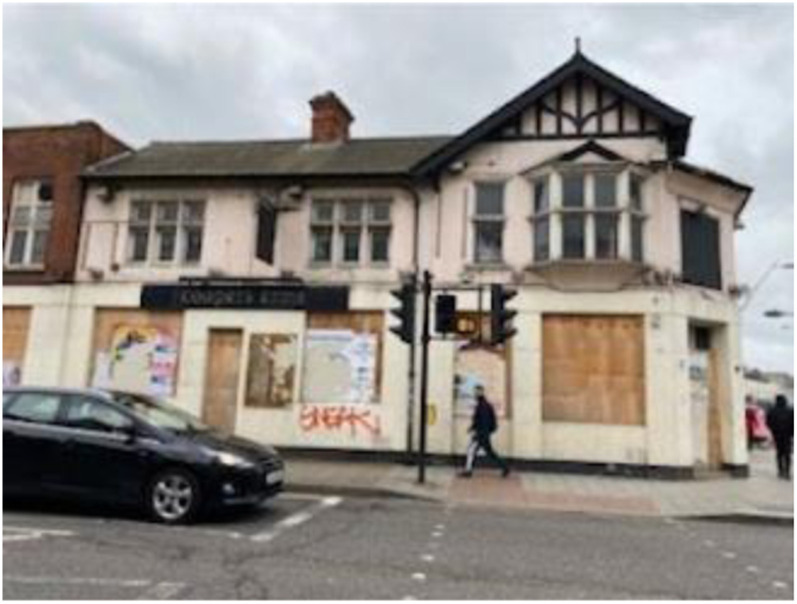


Photo 18, Participant 3

Furthermore, adolescents recognize disparities in the distribution of beautiful buildings across London, which “seem too good to be in East London” (Participant 7). For instance, Participant 3 describes how “more buildings, particularly where I live, are becoming run down and derelict.” This sentiment is evident in their photo (photo 18, left) containing a disused building where “the windows are broken in … it is now boarded up and destroyed and it makes me upset.”

Similar attitudes are repeated in Participant 3’s caption of a photo containing another dilapidated building. She writes:

This is The White Horse pub. It, unfortunately, was burnt down and destroyed by a gang a few years ago and has been left like this since. This upset me because I used to come here a lot when I was young and spend much time in “secret” gardens.

These remarks appear to corroborate with Howard (2016), who recognizes how buildings can evoke nostalgia, which Hepper and colleagues (2021) more recently recognize as a predator of happiness. Thus, the deterioration of building conditions, which affect parts of East London (Moore & Woodcraft, 2019; Pappalepore et al., 2014), may affect adolescents’ memories, potentially influencing their happiness and sadness.

The above evaluation highlights the complexity, often contradictory and intersecting nature of nature and the built environment on adolescents’ happiness and sadness. The insights from our research, grounded in context and theory, could contribute to developing new understandings and refining existing theories related to mental health and the social world. For example, our research helps to confirm the validity of Ulrich’s (1983) Stress Recovery Theory, underscoring its relevance in understanding how adolescents interact with the urban environment post-COVID-19.

Furthermore, our research supports the usefulness of the community-based participant methodology within adolescent mental health research. The participatory process likely promoted adolescents’ autonomy and reduced the effect of power dynamics between adolescents and researchers, potentially leading to more authentic insights. Additionally, the methodology’s underlying principle of inclusivity promotes a promising yet partial solution to the misrepresentation and exclusion of East London adolescents within mental health research.

Additionally, our study provides evidence demonstrating the feasibility of Photovoice within mental health research involving East London adolescents. Our use of Photovoice underscores how combining photography and narrative can engage adolescents in dialogue to co-produce mental research exploring determinants impacting their happiness and sadness. In turn, this knowledge can help identify the mental health and social needs of East London adolescents. However, the full engagement of adolescents in our study was limited by various factors affecting their lives. As such, other approaches should be considered alongside Photovoice to engage adolescents in mental health research.

### Strengths and Limitations

Some strengths and limitations of our study are discussed above and linked with specific findings. A key strength of our research is that it is the first in England to investigate social factors influencing East London adolescents’ happiness and sadness. We address the methodological and reporting limitations identified by Stephens and colleagues (2023) and have generated insightful and powerful findings that are emotive, metaphorical, and useful. Our findings, co-produced with the adolescents as participants, add to limited research on adolescent mental health, as noted in Fløtten and colleagues’ (2021) scoping review.

The study had limitations as well. Participants needed to own or have access to a phone to take part, which may have meant specific, deprived adolescents could not take part. In addition, all of our participants were recruited through a single organization and acquaintances of existing participants, potentially reducing the generalizability of the findings. However, FNoble and Smith (2015) consider generalizability to be an inappropriate concept in the context of qualitative research. This statement applies to our research, which aims to produce locally situated findings concerning a particular population rather than widely generalizable findings.

Our data collection coincided with spring and Ramadan, which may have influenced the participants’ experiences and perceptions of their environment. This context should be considered when considering the findings.

Strengths of our approach include actively engaging adolescents throughout every stage of the research process harnessing their expertise to co-produce relevant mental health research that reflects their lived experiences. Furthermore, mobile phone photography, a common technology used by adolescents (Toh et al., 2019), may have fostered adolescents’ engagement in our study.

However, as researchers and readers, we should consider whether photos adequately represent adolescents’ realities and the meaning they attribute to them. An interview study by Chua and Chang (2016) notes how adolescents can use images to promote social desirability bias. For instance, Ronzi and colleagues (2016) note how most photographs collected throughout their Photovoice study were of positive aspects, a trend apparent in our study. Thus, social desirability bias could have affected the authenticity of the data collected, analyzed, and presented in our manuscript. There is also a risk that participants shared what they thought the researchers wanted to hear: this form of bias was reduced by having the focus groups as participant-led as possible.

Future mental health research using Photovoice to examine mental health may consider ways to address ethical barriers that prevent the inclusion of photos of people in such studies. Enabling adolescents to collect photos of people may produce different findings to Photovoice studies restricting their inclusion and may promote a more complete understanding of adolescents’ mental health.

## Implications

Our findings reveal potentially significant insights examining determinants of adolescents’ happiness and sadness and reinforce a need to ensure adolescents feel connected to nature within the urban environment. Photovoice can impact new policy development and existing policies (Sanon et al., 2014), and our data, generated by adolescents, may inform various stakeholders in creating and improving policies and practices to safeguard adolescents’ mental health.

Our research demonstrates how various sectors and service areas determine adolescents’ happiness and sadness. By utilizing this information, specifically the finding that nature is embraced and dwindling, policymakers can improve the direction of the “Health in All Policies” (Public Health England, 2016) approach and London’s Environmental Strategy (Greater London Authority, 2018). Such changes to policy could improve the mental health and social needs of East London adolescents. Furthermore, our findings could inform future research in this field, helping to create better policies, services, and practices that cater to the mental health needs of adolescents.

## Conclusion

Our study used Photovoice to explore social determinants influencing East London adolescents’ happiness and sadness. Our research, in collaboration with the adolescents as co-researchers, underscores how factors, including environmental conditions and social networks, influence adolescent mental health. We identified four themes relating to nature and the built environment. Each theme provides a comprehensive and nuanced understanding of the factors influencing adolescents’ experiences of happiness and sadness. The insights produced by our research have practical applications, including informing policy, urban planning initiatives, and educational and therapeutic programs.

Notably, our research exemplifies the potential of Photovoice to empower adolescents to co-produce research, promoting awareness and cultural competency. Given the relative novelty of Photovoice in the field of adolescent mental health, our research is significant in further developing the method.

In summary, our research offers new insights into the social determinants of adolescent mental health and indicates that the use of participatory methods, such as Photovoice, affords a (literal) view that may otherwise have been missed. This study thus provides support for using inclusive and participatory methods. It contributes to the emerging body of evidence in this field and can shape the direction of future research and application of the Photovoice method.

## Supplemental Material

Supplemental Material - “Instead of Building More Buildings, They Should Plant More Trees”: A Photovoice Study of Determinants of Happiness and Sadness Among East London Adolescents Supplemental Material for “Instead of Building More Buildings, They Should Plant More Trees”: A Photovoice Study of Determinants of Happiness and Sadness Among East London Adolescents by Madison Stephens, Nargis Rahmanfard, Maev Conneely, Victoria Bird, Alec Knight, and Paul Heritage in Qualitative Health Research

Supplemental Material - “Instead of Building More Buildings, They Should Plant More Trees”: A Photovoice Study of Determinants of Happiness and Sadness Among East London Adolescents Supplemental Material for “Instead of Building More Buildings, They Should Plant More Trees”: A Photovoice Study of Determinants of Happiness and Sadness Among East London Adolescents by Madison Stephens, Nargis Rahmanfard, Maev Conneely, Victoria Bird, Alec Knight, and Paul Heritage in Qualitative Health Research

Supplemental Material - “Instead of Building More Buildings, They Should Plant More Trees”: A Photovoice Study of Determinants of Happiness and Sadness Among East London Adolescents Supplemental Material for “Instead of Building More Buildings, They Should Plant More Trees”: A Photovoice Study of Determinants of Happiness and Sadness Among East London Adolescents by Madison Stephens, Nargis Rahmanfard, Maev Conneely, Victoria Bird, Alec Knight, and Paul Heritage in Qualitative Health Research

Supplemental Material - “Instead of Building More Buildings, They Should Plant More Trees”: A Photovoice Study of Determinants of Happiness and Sadness Among East London Adolescents Supplemental Material for “Instead of Building More Buildings, They Should Plant More Trees”: A Photovoice Study of Determinants of Happiness and Sadness Among East London Adolescents by Madison Stephens, Nargis Rahmanfard, Maev Conneely, Victoria Bird, Alec Knight, and Paul Heritage in Qualitative Health Research

Supplemental Material - “Instead of Building More Buildings, They Should Plant More Trees”: A Photovoice Study of Determinants of Happiness and Sadness Among East London Adolescents Supplemental Material for “Instead of Building More Buildings, They Should Plant More Trees”: A Photovoice Study of Determinants of Happiness and Sadness Among East London Adolescents by Madison Stephens, Nargis Rahmanfard, Maev Conneely, Victoria Bird, Alec Knight, and Paul Heritage in Qualitative Health Research

Supplemental Material - “Instead of Building More Buildings, They Should Plant More Trees”: A Photovoice Study of Determinants of Happiness and Sadness Among East London Adolescents Supplemental Material for “Instead of Building More Buildings, They Should Plant More Trees”: A Photovoice Study of Determinants of Happiness and Sadness Among East London Adolescents by Madison Stephens, Nargis Rahmanfard, Maev Conneely, Victoria Bird, Alec Knight, and Paul Heritage in Qualitative Health Research
