# Supplementary figures and images for: “Instead of Building More Buildings, They Should Plant More Trees”, a Photovoice Study of Determinants of Happiness and Sadness Among East London Adolescents
Source: Qual Health Res. 2024 Nov 14;35(9):1068–90. doi: 10.1177/10497323241291667 (PMC12202830; doi:10.1177/10497323241291667)

## Social Identity Map

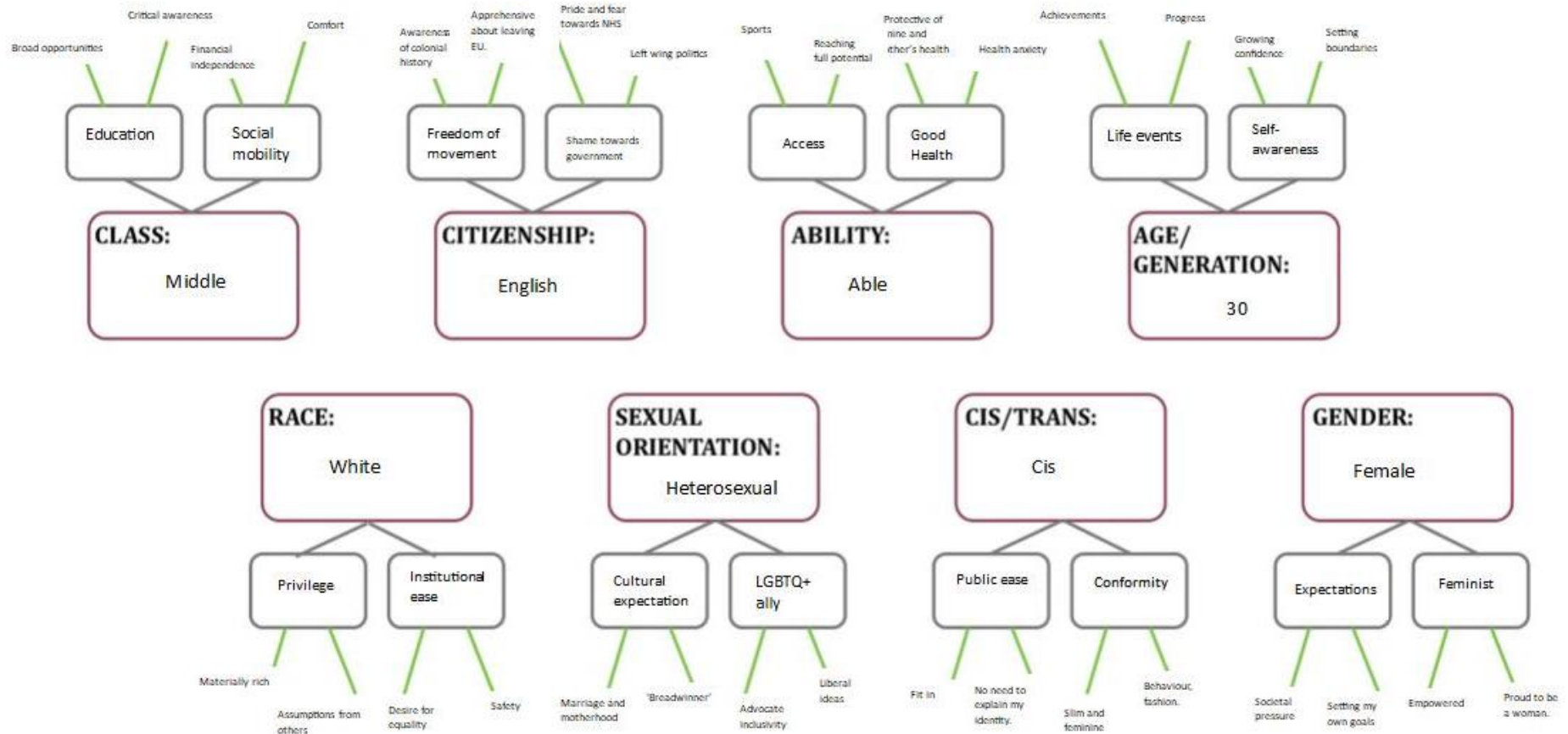

Supplement: Supplemental Material - “Instead of Building More Buildings, They Should Plant More Trees”: A Photovoice Study of Determinants of Happiness and Sadness Among East London Adolescents [file sj-pdf-1-qhr-10.1177_10497323241291667.pdf]

Social Identity Map - NR

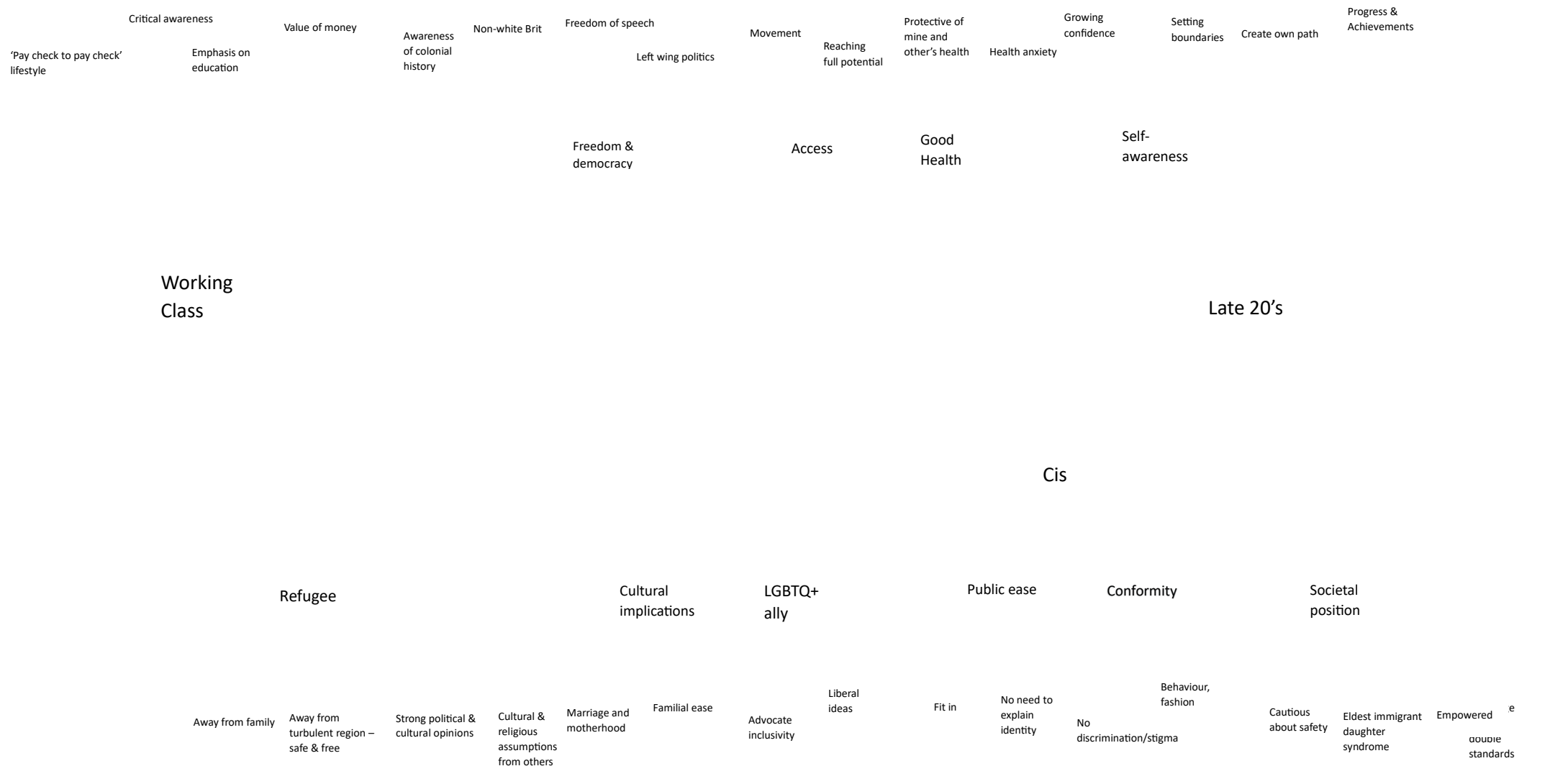

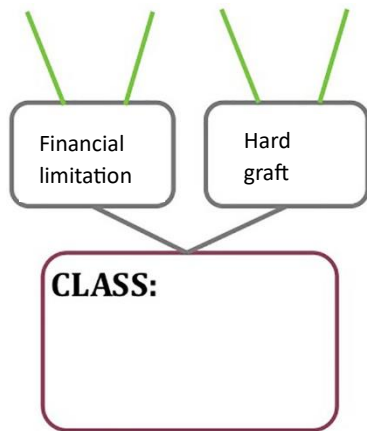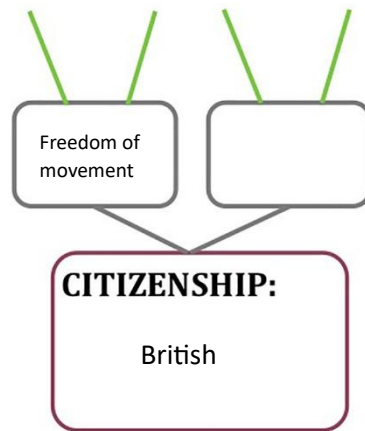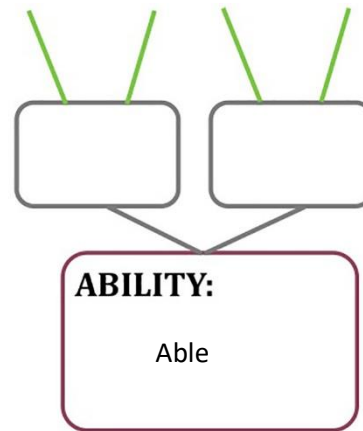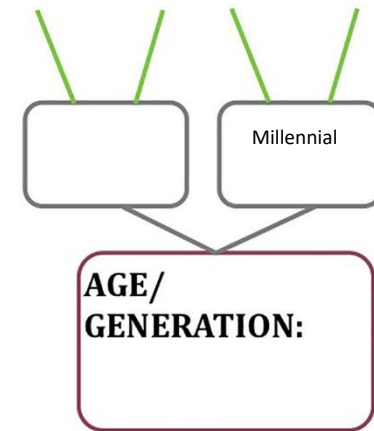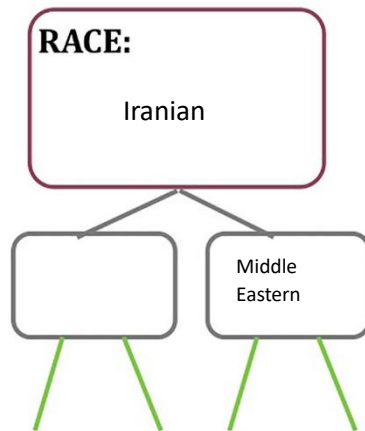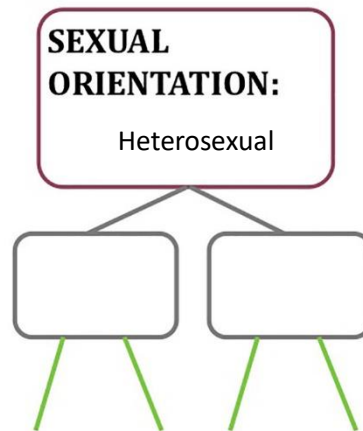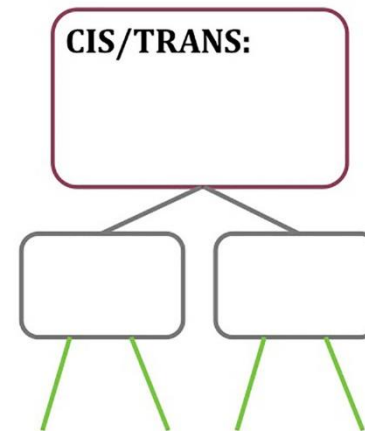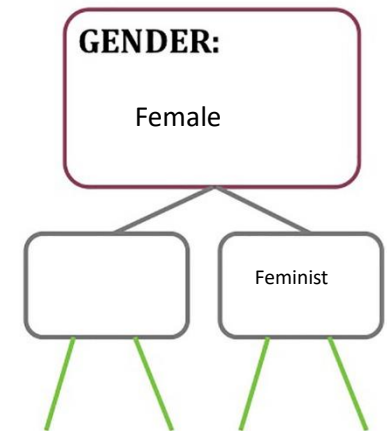

Supplement: Supplemental Material - “Instead of Building More Buildings, They Should Plant More Trees”: A Photovoice Study of Determinants of Happiness and Sadness Among East London Adolescents [file sj-pdf-2-qhr-10.1177_10497323241291667.pdf]
