# Supplementary material for: “Instead of Building More Buildings, They Should Plant More Trees”, a Photovoice Study of Determinants of Happiness and Sadness Among East London Adolescents
Source: Qual Health Res. 2024 Nov 14;35(9):1068–90. doi: 10.1177/10497323241291667 (PMC12202830; doi:10.1177/10497323241291667)
Supplement: Supplemental Material - “Instead of Building More Buildings, They Should Plant More Trees”: A Photovoice Study of Determinants of Happiness and Sadness Among East London Adolescents [file sj-pdf-3-qhr-10.1177_10497323241291667.pdf]

SOCIODEMOGRAPHIC INFORMATION FORM

Where applicable, please tick the ONE accompanying box that is the most relevant

1. Age (in years) ☐☐

2. Gender
   1. Female ☐
   2. Male ☐
   3. Non-binary ☐
   4. Prefer not to say ☐
   5. Other (please specify below) ☐

   ____

3. Country of birth — Country: ______________________________

4. First language
   English ☐
   Other (*please specify below*) ☐

   ____

5. Ethnicity (*please write most relevant number in the box*) ☐

White

1. White (English, Welsh, Scottish, Northern Irish or British)
1. Irish
2. Gypsy or Irish Traveller
3. Roma
4. Any other White background

Asian or Asian British

5. Indian
6. Pakistani
7. Bangladeshi
8. Chinese

9. Any other Asian background

Black, Black British, Caribbean or African

10. Caribbean
11. African
12. Any other Black, Black British, or Caribbean background

Mixed or multiple ethnic groups

13. White and Black Caribbean
14. White and Black African
15. White and Asian
16. Any other Mixed or multiple ethnic background

Other ethnic group

17. Arab
18. Any other ethnic group (Please specify below)

____

6. Occupation of parent(s)/legal guardian(s)

____
