# Supplementary material for: “Instead of Building More Buildings, They Should Plant More Trees”, a Photovoice Study of Determinants of Happiness and Sadness Among East London Adolescents
Source: Qual Health Res. 2024 Nov 14;35(9):1068–90. doi: 10.1177/10497323241291667 (PMC12202830; doi:10.1177/10497323241291667)
Supplement: Supplemental Material - “Instead of Building More Buildings, They Should Plant More Trees”: A Photovoice Study of Determinants of Happiness and Sadness Among East London Adolescents [file sj-pdf-4-qhr-10.1177_10497323241291667.pdf]

Respect and kindness guidance

- Participants should not discuss who has captured which photographs within and outside of the focus groups.
- Participants should not discuss the conversations had during the focus group outside of the focus group.
- Participants are accountable during the collection of photographic images and should not take photographs of people where they can be easily identified or of incriminating activity.
- Participants should ask questions if they are unsure of the process.
- Participants shall show respect and kindness to others in the group so discussions are held in a safe and empowering environment.
- Participants will listen to their peers and take turns communicating.
- Participants shall inform the lead researcher if they encounter any difficulties that prevent them from participating fully.

Signature: -

Name: -

Date:
