# Supplementary material for: “Instead of Building More Buildings, They Should Plant More Trees”, a Photovoice Study of Determinants of Happiness and Sadness Among East London Adolescents
Source: Qual Health Res. 2024 Nov 14;35(9):1068–90. doi: 10.1177/10497323241291667 (PMC12202830; doi:10.1177/10497323241291667)
Supplement: Supplemental Material - “Instead of Building More Buildings, They Should Plant More Trees”: A Photovoice Study of Determinants of Happiness and Sadness Among East London Adolescents [file sj-pdf-5-qhr-10.1177_10497323241291667.pdf]

Transcript of focus group

1. The first one we're going to start with is this picture and just for the interests of confidentiality, please don't shout out if it's your picture. We're just gonna have a conversation. So if everyone wants to kind of slide that one along. This is picture of a fountain. And the first question here is whaclt do you see? And if everylone kind of talks kinda of out loud, but just being mindful of giving each other space to talk. So what do you see here?
2. Um I see like tall buildings in the background behind the trees.
3. I can see that it's a good weather outside.
4. Um I can see mostly nature and just like the water flowing.
5. I see water
6. You see water
7. Um, I see water in like the middle of a park with like surrounded by trees.
8. The area looks like it's looked after.
9. Yeah, I agree. It looks like a well kept area with the trees and the water
10. OK. And the next question about this picture is what is really happening here?
11. It looks like people are going for like walks. I would guess it looks like an area where someone might walk by
12. Yeah, it looks like a communal area. You can see people sitting on the benches and walking past
13. It looks like a path with like loads of people and like the weather is really nice.
14. What was the question?
15. So the question is what is really happening here?
16. Um, It seems like an area where people come to look at some nice shoes and perhaps for some peace and quiet.
17. It looks like a park and the weather is obviously really nice.I think people just taking advantage of it.They're just... yeah.
18. I think people are just walking around and enjoying the nature around them.
19. Yeah, I think people are, like, admiring it because it looks really nice
20. And the next question and with all these questions, there's no right or wrong. So how does this relate to our lives.
21. Umm it might show how we like sometithere mes take the time to look at something and appreciate it.
22. Nature is really important because without nature, we won't have a clean air to breathe.
23. Umm I feel like they don't really appreciate the things around us and just don't take it. Don't take the time to actually see what's going on. And I think that there's definitely some people out there that do care about nature. That's why they care enough to actually make sure that the area is clean.
24. Uhh, It seemed like a utopia of some sort, and it relates to my life because I study Utopia in school. So then if I think about an image of what looks like a perfect world, this would be my perfect world expect ... like if there was no humans and just this.. It would be a perfect world
25. Um, wait What was the question again? How does it relate to your world?
26. That’s okay, so the question is how does this relate our lives?

27. For me, I think it it's like.. When everything's so busy, we don't like look around to take in the little things. So like it’s a park with like loads of people, but a lot of people walking by and they don't really like stop to look quiet. That's my perspective of it.
28. I think it's important because it kind of reminds us to take a moment of silence and
29. just appreciate and think
30. OK. And then..
31. Oh. Um. Mm
32. So how does this relate to our lives?
33. Umm. It kinda looks like an escape area, so like just somewhere where someone can go and like some quiet and You know if like stressed or have something on their mind, just like a place to escape.
34. OK. And the next question is why does this condition exist?
35. Sorry, what do you mean by that?
36. Umm ...So, why? Why. Why does this exist? Again, there’s no right or wrong answer with that one
37. I think it's just like a place like. Like in a park where people can just like look... and just a nice place to sit around, Just like a different... a different like setting
38. Umm it looks like it's in a pretty like urbanized area, so it's probably to break up the the buildings by having a little bit of greenery and allow the people who live in the area to enjoy it
39. Umm I think it exists to like give people a break because like there's loads of like tall buildings and everything, so it could be like a place where people just like sit down to like, take a minute to breath.
40. Umm, because the Council wanted nicer placed to look at
41. I think because … just like the term littering.. littering, is a lot more popular now. So the Council, the government wants to do more about it because it's gonna affect us in the future. So that's why they make places like these to kind of make people be more mindful of what they do.
42. Umm. This area looks like it's well looked after because I don't see any litter and it looks really clean.
43. Umm I feel like it’s there to like bring peace and like calmness to like people's busy lives. Maybe
44. OK. And then the next question to circulate back around is what can we do about it?
45. Umm, we can carry on like enjoying the space because it's built for the public and they should like take time and like step away from their busy lives and like, be calm here.
46. Umm, I think instead of building more buildings, I think they should plant more trees and encourage more nature.
47. Um we could preserve more. We could preserve areas like this a lot more. So we see it a lot more around us.
48. Use this as an example of what type of areas we should have in more like localized places and stuff? I think that it should be used as an example to create more spaces like this for people to just like get out from the world and just like go to their own little world.
49. I think they should maintain places like this, so like it still exists in the future and like it stays in condition it’s in like clean and like not dirty and stuff.
50. We can encourage parks like these to be the standards for all areas within like umm Like as populated as this place or not?

51. Umm I agree, I think more place like these should be set up because it looks like people are appreciating it by walking by and sitting down and watching,
52. OK. Brilliant. So the next picture is going to be the one of the school.So I think If we start this way around. So.. what, what do you see here?
53. Umm I see students and I think they're queuing up.. not sure.
54. Umm It looks like it's the school's common room where everyone um.. This is like um a quiet place where you can do your homework.
55. Umm, It is, but, but what she said... it does look a common room and I feel like.. It's probably a place for everyone to just calm down a bit from everything they've done for the day and just talk about anything they want with their friends.
56. Umm It looks like it looks like some sort of classroom where people are just like chilling, going on their phone so I'm guessing you're allowed to go on your phone, so I'm guessing it's just some like socializing space.
57. Umm it kind of looks like a canteen where, like people sitting and, like, waiting for food, maybe.
58. Uhh, Yeah, I agree. It does look like a canteen and maybe like a communal area where students go to during break or lunch
59. Yeah, I think it's like a communal area, just like people interacting, just relaxing or like during lunch or something,
60. OK and then this next question, what is really happening here?
61. Umm I think like, like the students like talking each other, talking to each other, and like interacting with one another.
62. Umm I think the students are waiting to get a seat.
63. It looks like these students are just.. yeah, they're just talking to each other.
64. Umm...basically yeah, it looks like people are just like talking and trying to socialize or something, or waiting to get a seat or just.. I don't..yeah, so just think they're just hanging out with their friends.
65. Umm, I think they're like on their break, umm waiting for something or like these people in the back talking and sitting down.
66. Umm, yeah, I think it's just a place where friends meet cos there's a group at the back and there's also like Macbooks, so it might be like a learning area as well.
67. Um I think it's like a chance for friends to get together and hang out together.
68. OK. And we're going to go back around this away. How does this relate to our lives?
69. Umm I think all of us, most of us, I don't know... are at school so, um I can assume that a lot of us have places like this in school so probably been in places like this and done some of the things that people are doing in this picture
70. Yeah, it looks like it's in a sixth form or a college, so it can kind of relate to how.. I don't know, the adolescent experience there maybe.
71. Umm This looks like a typically like break or lunch time, when like everyone's just like having a break from lessons. So, like our everyday lives, if you go to school and stuff.
72. Umm it relates to my life because we have a common room in my 6th form. It's not as big as this, umm but it's the time to like. It's socializing my friends.. Especially when the weather is bad outside and I don't...
73. [Audio inturrupted by arrival of participant] "He arrives...come on in"

74. Think most schools provide their students with a space for them to talk to each other and just give them a break from everything they’ve been doing for the whole day.
75. I can relate to this picture because in my school there's a big common room like this where I'm I'm able to meet my friends and also ask them help with um my studies.
76. Yeah,I think like everyone can relate to just like talking to their peers during break or studying.
77. Okay... and then. [redacted], I’ll get you up to speed on the next one.
78. Okay
79. So the next question about this picture, ...Can I grab that one? Why does this condition exist? And Again, there's no right or wrong answer
80. Maybe just like as a place for students to take a break from like being in a classroom setting.
81. Yeah, I think it's a place for students to relax and maybe center themselves again after hours of learning.
82. Yeah, I think it's just like a place for everyone to refresh themselves and like just take a break from everything
83. Umm yeah I think,, this condition exists so that people can socialize and actually engage people with people own age rather than just like constantly studying and out of proportion.
84. Umm, it helps students to just relax for a bit because they've obviously got stuff going on with their studying and yeah, just help them to relax.
85. Um I think this condition exists because it allows students to um get um a move away from exam pressure and stress.
86. Yeah, I think it allows students to take a break.
87. OK. And then if we go back around again.So what can we do about it?
88. Maybe we could increase opportunities like this for young people in all schools.
89. Um, all schools have a common room like this, so make sure that every school has a common room.
90. Have more spaces like this so that more people can use it and..yeah
91. Umm I think that whilst it's useful having a space where everyone can socialize, there needs to be a place where if you don't wanna socialize that you have a similar room like this big with the space that is in here but for people that don't, really want to be with everyone else.. Just some quiet space and stuff
92. Sorry what was the question?
93. The question is, what can we do about it?
94. Umm, provide more spaces like this and um.. Yeah, just like, make sure that everyone has, like, a place where they can, like, take a breather from...
95. We can encourage spaces like this to be as spacious and large as it is, because it doesn't seem very overwhelming or like over-stimulating for students. Whereas some common rooms can be quite small.
96. umm, think, umm.. Should encourage like students to um go to these places to take a break from the pressures of school.
97. Okay brilliant. So [redacted], we are just going around and we're going to be talking about each picture and going through a set of questions. Umm but we’ll go back to the once you have missed and make sure you have had a chance to say everything. So next one will be umm, the sunset one which I think is close to [redacted]. So Iif I start with you [redacted]. So what do you see here?

98. So I believe this is a park. Then umm this is during like sunset hours.
99. Yeah, I think its like some kind of like big field
100. Yeah, it looks like a field with those gates around so.. I don't know maybe in a gated area
101. Yeah, it looks like a field umm during sunset.
102. Yeah, it looks like um a big park and it's sunset hour and people are just like watching it.
103. Um, Yeah, looks like a park and there's gates around it so there's probably a place to play sports like tennis and badminton or anything like that. And there's also a sunset
104. Um It looks like a park where people can relax and also play sports.
105. Um yeah, it looks like a field and there's like houses in the back.
106. OK. And then going back around again, so what is really happening here?
107. Um the sun is setting, and like the day is ending.
108. Um It looks like the afternoon.
109. Um there's a sunset.
110. From my perspective, yeah, I feel like what's really happening yeah is that someone just wants to escape their mundane life, and they need to see something more beautiful and so they watched the sunset, which is quite stunning.
111. Umm It's really... it looks really secluded, like no one's there and the sun's setting so it's nearly like the end of the day
112. Yeah, looks like maybe the park is closing soon and the person taking the photo, it's one of the last people there appreciating in the view.
113. Umm I think that because the sun is setting and it's like getting late that if it was a park then people have left and probably that's why it's empty.
114. Yeah, so um the suns going went down and it just shows like it's the most relaxing part of the day..in my opinion.
115. OK. And how does this relate to our lives? Start with you [redacted] and go back around
116. Yeah, I feel like it's relates to our life because it's like symbolic of how..the suns... during the sunset we get more tired and we actually go down as well. So everyone has like a nine to five and then during these times we tend to like sit down and relax and the same things happening here with the sun.
117. Umm I think that a lot of people like to appreciate the sunset and I think a lot of people can agree like it's... it's a nice thing to to watch.
118. Yeah, I think it's just a reminder to relax and enjoy the like the scenery around us.
119. Umm yeah, so sunsets like this doesn't happen every day so it's like a reminder to like just look around and like take a break from everything.
120. How does it relate to our lives?
121. Happens every day. So like, I don't know, it's just part of our everyday lives.
122. It relates to my life because I love sunsets and like um my friend, every day he takes a picture of the sunset for me because he knows I love them, so he takes it around for me. So it's like a very It's very important aspect of my day because I love watching the sunset.I just love it just so it's such a calming time and just to reflect on stuff
123. Um of course sunsets happen every day But I don't let people appreciate the things around them.So that's why I think it's important to just take your time, take a bit of time for yourself to see what's actually happening around you and just be able to relax.

124. When I'm overwhelmed with stress, especially during a-levels, I always look outside in the window and see the sunrise and it helps me like calm down and also not think about exams.
125. Yeah, I think it's like a reminder to reflect and relax.
126. OK.And going back around with this one. Um What can we do about it?
127. I think we should take opportunities like this every day and remind ourselves to like, stay calm and..relax,
128. Just appreciate the sunrise and just take the time.Take take time off of your day and just look at the sunrise.
129. You should always leave a bit of time for yourself... Just kind of relax yourself when everything has happened on the day.
130. I think that we could continue to appreciate things around us and to do things that really work for us...If looking at the sunset really works for you and it helps you then continue doing it.
131. I think we could all like appreciate the little things and like, look at what's surrounding us.
132. I think it should be taken as a reminder to appreciate the nature that surrounds us and really just...
133. Yeah,
134. We can take it as an opportunity to like wind down like at the end of the day.
135. I think we should like synchronize with the sun as the sun is going down, we should start dropping our daily activities and start relaxing as wel.l And, um, yeah.
136. That's brillaint great. [To redacted] and do you want to introduce the next one? [redacted] could you pass me that one please
137. So we're just gonna, I'm just gonna ask you all of you what do you see here?
138. OK, so I see that there's traffic just cars going by during the normal day.
139. Just looks like cars driving on the road, maybe it's busy.
140. I see like an abandoned pub and in the roof part it's been burned down.
141. It looks like the middle of the day with like cars driving around.
142. Yeah, it looks like some abandoned,..not abandoned cos there's cars driving.. it looks like... It just looks looks like a space where people are driving past a building that looks quite run down and not in use anymore.
143. It looks like a busy road because you can obviously... there's ..you can obviously see that there's a lot of cars there and I think it's just a regular day... Person's life.
144. Um It looks like a really busy road because there's a lot of cars there
145. Umm it looks like a road with cars driving in both directions,
146. OK great. And now what is really happening here?
147. So what's happening here is that there's practically traffic everyone's going... looks like during like 4 to 5:00 PM So everyone's probably on their way back home from work.
148. It just looks like a normal day. People driving to places that we need to be.
149. Yeah, it seems like people are focused on getting either home or to a place that
150. they need to be.
151. Yeah, it just looks like the middle of the day and like everyone just trying to get the place that they need to be.

152. It looks like, yeah... People are driving home in nice cars, you know. [laughter] And yeah, they're driving past the building and they're in a rush...But they're leaving gaps while they drive so it's good, safe driving.
153. It looks like the middle of the day, so there's people probably coming back from their jobs from a busy day.
154. It looks like people are going back home from their jobs.
155. Yeah, mostly looks like people are going home or where they need to go
156. Brilliant.
157. And how does this relate to our lives. So if we start again
158. This relates to our lives because.. I don't know...I personally drive so... um traffic it adds up to the time taken to get to your house.
159. I think we might know people that drive or drive to work or school.
160. Yeah, I think it relates to like the busyness of life and how we can kind of lose focus on what's important.
161. It relates to my life because everyone in my family drives, so I'm normally in a car going somewhere, so it just looks like our everyday lives.
162. It relates to my life because when I'm stressed, I like going on long drives and I like looking out the window and just observing what I'm going past.
163. It relates to my life, because.. Because. It relates to my life because there's always people out there driving to whatever they're meant to be and it's kind of relaxing, relaxing to see people just minding their own business I guess.
164. It relates to my life because both of my parents drive so whenever I want to go somewhere I'm always in the car, but I hate being stuck in traffic and especially in East London.. there's always cars everywhere
165. Um I think it shows that we always have like a place to be.
166. OK. And why does this condition exist?
167. This condition exists due to the high population in London and because everyone has cars there's a lot of congestion.
168. Umm I think a lot of people, a lot of people owns cars, so it just makes it easier for transport.
169. Umm, I think the condition exists because..more buildings, particularly where I live, are like becoming run down and derelict. So it might condition us to.. I dunno help restore these buildings that are in our surroundings.
170. I think exists because a lot of people own cars.. so like ...it's just like a quick easy way to like get from one place to another.
171. This condition exists yeah because people prefer driving than taking public transport for obvious reasons and like... yeah and I think sometimes societies will, like, socialises us into believing that the best way to do things is the easiest way to do it rather than taking the longer route. Yeah, I think this is just a product of society telling us that driving is the best way to go forward.
172. Sorry, what's the question again?
173. Why does this condition exist?

174. It exists because it's probably the most convenient way for people to travel...They like to..they like the easiest way to get to places instead of for example using public transport, which obviously takes longer.

175. I think this condition exists because most people travel together as a family and it's easier to travel in by cars than public transport which takes a long time.

176. I think this condition exists because it's the easiest and most safest way for most people to travel.

177. And what can we do about it?

178. I feel like we can use other forms of transport such as trains, which can cut the amount of congestion.

179. Yeah, I agree, I think we should try to use other transport.. to like.... The air pollution is likereally bad and like having.. driving Cars contributes to that.

180. Yeah, I think we could try and make areas look more aesthetically appealing so that people want to walk and take other forms of public transport instead of just driving and trying to get out the area as quickly as possible.

181. Yeah, I think we can like take alternative ways instead of like always taking a car so if you're going somewhere quite close, instead of driving to even like walk or take the bus maybe.

182. I think that we can get people to encourage people to do other stuff apart from driving and stuff and I think that like... we know driving is not great for like air pollution and all of that so I think we should have more research out on that and, like, publicize it more so that people know the effects that driving actually has on the environment.

183. We should encourage people to use different modes of transport, such as public transport and also walking because it's just better for the environment and it brings down carbon dioxide emissions.

184. I think you should promote public transport because due to inflation, some people might not be able to afford public transport so lower the cost.

185. Yeah, I think we can use different modes of transport such as like walking or a bike.

186. Okay. So the next one is....this one, so one of the skyline. It's very pretty. [redacted] if I start with you, what do you see here?

187. I see a beautiful city view with the.. with the River Thames and the clear skies.

188. Yeah, I see some kind of body of water.. like buildings in background.

189. Yeah, it looks like the persons taken the photo on the dock, so maybe in the London dockland somewhere.

190. Yeah, it just like an overview of like all the buildings.. and like the O2 there

191. I... what was the question?

192. What do you see here?

193. Oh yeah, so basically yeah I see someone.. I don't, I don't think they're on the bar I think it's that place where you're standing waiting on the dock yeah. Waiting to get on the boat and stuff and someone saw a very beautiful.. time to take an Instagram worthy photo [laugh] and they did it and stuff. And it's during the night, so I'm guessing they're coming back from somewhere or they're going somewhere?

194. I can also see a dock and a body of water, and the person is probably just taking your time to look at the buildings around them.

195. It looks like a picture taken at night so... and I can also see houses and tall buildings in the background.
196. It looks like a night time picture of the river and the city.
197. OK. And going back around again. What is really happening here?
198. So it's showing how.. personally showing how London's always active, how through the... Lights on in the buildings... shows that people are never asleep and the city is always alive.
199. I think that because it was taken like at night.. looks like someone just tryna find a quiet place?
200. I think building off the city life point, it's even though it's dark and it's obviously maybe a time when people would wind down. There are so many lights on and it looks like either a town block like a flat block or workspaces which are still completely on...So maybe people are still working.
201. Yeah, there's lots of like lights on like this house is there.So, like, maybe like everyone's going home and, like, ending their day.
202. I think that.. it's kind of reinforcing the idea of what's already been said that London does not sleep because there's so many lights on. And even though it's at night like.. Even though it's night, you can clearly. You can actually see like on like.. based on what you see, you can actually see a lot of meaning behind London and stuff. I mean, it's literally a picture that speaks 1000 words because you can see ...you can make so much meaning out of what's going on in front of you and stuff
203. It shows that London has a very active nightlife. So even though it's really dark at night, there's always people out there that are doing are doing something important, I guess.
204. Because there's so many lights on, it also creates a safe environment for you to walk around and it shows that London is always on.
205. Yeah, kind of like shows like the city is like awake...And even though it's like night time, no ones going home yet.
206. [To redacted] did you answer that one?
207. Yeah I've answered that one
208. And then the next one is how does this relate to our lives?
209. This relates to our lives because... well the street lights...so it gives us a form of direction and awareness in the night
210. It relates to my life because I live In London so there are places like this to see all over London
211. Yeah, I think it's it relates to my life because it shows that even though this might only be 20 minutes from an area that I live in, like in East London, this looks like it's closer to central, that we're still very close to.. the nightlife and being able to enter the city and be a part of it .
212. This relates to my life because it's kind of close to where I live and...Yeah, the buildings are just .. a lot of places around London they look like that.
213. It relates to my life because I think it's such a great way to, like, spend time with your friends. Cause like I've gone with my friends to like, go and see the view and stuff and I think that it's just a great way to escape.
214. It relates to my life because it kind of just shows how chaotic and busy the city can be.
215. It relates to my life because it's a place where me and my family go out to spend time together.

216. It's to my life because it's like an area where we could easily go because we live in East London.
217. And if we go back this way. Why does this condition exist?
218. Umm think because it's London and it's like a city, like a large city with a lot of people, everyone is like awake. Everything is on.
219. I think this condition exists because like due to industrialization, there's more opportunities for people to work, and now there's like 24 hour services. So most buildings are always like in operation.
220. What was the question again?
221. The question was, how does this relate? Sorry, no, it's not.Why does this condition exist?
222. I think London is known to be a very busy city because there's a lot of people that travel from the outskirts into London because that's where most of the main buildings are.. so it kind of just shows that people work late at night and it kind of just pays off at the end because...
223. I think this condition exists because it's just another way to get somewhere, it's.just a better, fun way to get somewhere and a more beautiful way actually to get somewhere.
224. This condition exists because there's a lot like London's really populated. So like even though there's like you're living your own life. There's like people everywhere living their own life, like in their own houses and stuff.
225. I think this condition exists as a result of like the regeneration and gentrification happening in London right now.So even though it has like deindustrialization aspects, it's kind of using those elements which were made for manufacturing now in a more social like housing way.
226. Think that because it's in London on the Thames there's gonna be a lot of docks
227. This condition exists because of people's busy lives. I feel like because of the lights being on it shows, how a lot of people have... are overburdened with a lot of work.
228. And if we go back around this way, what can we do about?
229. I feel like we can embrace the.. the nature and the body of water that's there..that's a calming effect and not take things for granted.
230. Think we can appreciate and avoid...can you say littering in the Thames ?
231. Yeah,
232. Throwing like things that shouldn't be in there.
233. What was the question?
234. What can we do about it?
235. I think we can kind of embrace the way that this place has got older features of East London and it's combining it with the newer features. So instead of getting rid of like the history completely of an area, you can embrace it.
236. Yeah, I think we can just like take the time to appreciate stuff around us and like... just look around the area.
237. I think that we can visit these places more often and make most of them whilst they're still there.
238. If these actually are businesses that are working, we could appreciate the hard work actually does pay off at the end and that it's gonna be rewarding.
239. We should appreciate the nature around us and like she said, avoid littering.

240. We should like keep a balance like with the nature of the river and the city and the nightlife?

241. The next.... is everyone OK? Anyone need a comfort break?

242. [General murmur]

243. Yeah, you're all good? So the next one is this one is..... this one. Which is the book, so if we start. [redacted],if we start with you. So what do you see there?

244. I see a textbook with some colouring pencils and like stuff on the desk.

245. It looks like a sociology textbook.

246. I see a textbook on top of a table, but different pens and also books at the back

247. I see somebody really suffering cos of A-levels... and a textbook that is... what is this? Ohh method..sociology textbook.. and someone trying to study.

248. Yeah, it just looks like a sociology textbook. Research methods maybe.. In education

249. I see someone showing how intense textbooks can be sometimes with like hundreds of lines to learn.

250. I see a textbook..Maybe revision resources in the background

251. Yeah I just, I just saw.. see someone struggling with the amount of content you have to learn.

252. Okay and what is really happening here?

253. So, someone is like studying really hard and then trying their best to do well in the exams.

254. It looks like the person taking the picture is overwhelmed by the content.

255. I think the person is trying to study very hard and is kinda overwhelmed by the content, because you kind of you can see that this is kind of messy.

256. What's really going on here is that somebody is really trying hard for their exams and they are overwhelmed by the amount of content that they have to learn and the fact that it's not just one topic, it's just too many. And somebody really give up their social life for their exams.

257. It looks like someone's trying to study and looks really stressful and like overwhelming.

258. Yeah, it looks like someone's trying to study. There's a bunch of books at the back with, like, looks like marked papers and maybe stuff to recall and look back on.

259. Looks like someone trying to prepare for upcoming exams.

260. Yeah, it looks like someone's trying really hard to write for their upcoming A- levels.

261. And how does this relate to our lives?

262. Well, this relates specifically to the people on the tables lives because we have our upcoming A-levels and it's it's what we do, what makes most of our day.

263. I think a lot of people here either have a-levels or end year exams so I think I think this is the things I think people will probably be doing around this time.

264. I think people can relate to how stressful and overwhelming textbooks can be, this looks like a A&Q textbook. [laugher] So it'sprobably just filled with so many lines which are going to come up and maybe we just won't come up at all because they tend to do that [laugher]

265. It relates to our lives because we all like if you do a levels and stuff it'll relate like.. You use them to study and stuff and especially like when you're trying to revise for an exam you try and memorise everything.

266. It relates to my life yeah because I have upcoming a-level exams coming up, but also the fact that the person who made this text book yeah really thought were robots, [laughter] right? Like you could learn this whole thing but clearlyd not.And stuff and I think that it kinda .. it also relates to the fact that schools really do undermine us because they make us do all this work, but they also don't appreciate how much hard work actually goes into us obtaining results. It's not like we wake up one morning and we have an A-star you know we work hard and we revise six hours and stuff.That's how it relates to my life.

267. It relates to my life my life because I've got exams coming up soon. It can be kind of difficult to just fully understand the topic, because exams can do us dirty sometimes. The marks seem very specific.. which is not fair..sometimes.

268. It relates to my life because every subject requires a textbook and sometimes textbooks can be overwhelming with too much information that you don't really need in your exam.

269. It relates to my life because I also have exams I'm doing right now and it can be really overwhelming to see like so much text on a page and not being able to understand it straightaway

270. And back the other way. So what can we do about?

271. I think schools should put less pressure on us and that the exam system maybe it should be like changed a bit to kind of fit like all the students needs.. Like help with stress

272. Try and break down the information and use revision resources.

273. I don't really think schools have anything to do with exams. I think it's the government that think we're capable enough to do these types of stuff....But when they ask to do.. To do it, they can't do it. So it's kinda unrealistic expectations of 16 to 17 to 18 year olds

274. Tell this person don't pick sociology and don't encourage anyone else to pick sociology and tell their children not to pick sociology... Just don't pick sociology.

275. I think instead of like getting overwhelmed and do everything like take it.. in like chunks and like do it chunk by chunk instead of like going all over it all at once

276. Yeah, I think it can kind of showcase how much pressure 16 17 and 18 year olds are really put under nowadays. With content, which probably won't be relevant to any other thing in our lives, but we need to learn it for one exam which represents everything that we've done in those last two years, which I don't think is that good of a representation.

277. Probably taking a break from this stressful environment, especially with a really chunky textbook with lots of text in front of you

278. Yeah, you could try a different form of learning the content such as tutor to you, which is more simplified.

279. And I think the final question which I actually missed was why does this condition exist?

280. This condition exists due to the education system that the government put in place that everyone practically has to abide by.

281. I think it's that because the person has chose to revise for the exam so they can have the best possible outcome.

282. I think this condition exists to encourage students to read and kind of understand the content.

283. I think this condition exists because someones trying to revise to get like the best grades they can and to learn the content for the exams.
284. I think this conditioning is cause some genius came up with the topic of sociology. [laugher]
285. I think there's some unrealistic expectations for students to just understand everything the second that they're being told so, and it's not true.
286. I think this condition exists because I think the person is trying to do well in their exams
287. I think it exists so that the person can get good grades.
288. Okay. Brilliant. And then the penultimate picture is this one. So if we start with [redacted].. So what do you see here?
289. I see like a temple.
290. I see a temple and a person praying outside.
291. Yeah, I see a place of worship and people going inside it I think
292. Well, I don't think... so I see a temple. Yeah that's [incomprehensible]
293. Yeah, it looks like a really nice temple with like not that many people around.
294. Yeah, it looks like a temple, which may have been newly refurbished.
295. It's like a well kept place of worship.
296. Possibly a religious place of worship
297. Okay, and back around..so what is really happening here?
298. What's happening is that people may be going inside to perform their daily prayers from whatever religion or background they come from.
299. People probably go here to appreciate their religion.
300. Yeah, people seem like they're about to enter the place of worship.
301. Yeah, looks like a place where people go to worship and there's not that many people around.

302. Umm what was the question?
303. What is really happening here?
304. Someone is going to pray or actually admiring how beautiful this temple is and how well kept it is.
305. It's a place that helps people be closer to their religion.
306. People are going there to pray to God and also meet other people in the same community.
307. Yeah, I think people are like looking at the outside and maybe going inside to pray as well
308. And then the next question is, how does this relate to our lives?
309. I think cos lot of people are religious, like might reflect how they go and like, pray and worship.
310. I always pray to God when I'm going through something.. a hard situation or exam stress and it really helps me.
311. It helps people relax on whatever they're going in their lives because they know that there's always someone that's gonna listen to them... even though they're not physically there.

312. It relates to my life because I would pass it. Every day to get home from school. So I always see it. And I always sees like, umm.. 't's always really busy actually. So it's like and actually when I walk past it, it's one of the biggest buildings I walked past on my route home from school and stuff, but also, I'm not particularly religious, but I also like.. like to see how people like also are so, like, admiring to their religion and actually really committed?

313. Relates to my life because a lot of people are religious or follow a certain religion, so it's like a place to go back when you're feeling like stressed or overwhelmed.

314. What was the question?

315. How does this relate to our lives?

316. I think it relates to adolescents in general, because it's a place where people who are like stress, because of exams and stuff can just take a moment to appreciate like the community around them.

317. I think that there are a lot of places of worship in East London where we live so I think a think a lot of people go to visit as like a chance to escape.

318. Was the question.. what was really going on here?

319. How does this relate to our lives?

320. Ohh, this relates to our lives because we have different forms of like temples and buildings as well.

321. So it's really important to connect with your spiritual self once you enter these buildings.

322. Okay. Back the other way, what can we do about?

323. We can actually admire the beauty and the different religions in in this multicultural society.

324. Can respect the area as well as other people's beliefs and places of worship.

325. Think we can encourage areas like this to kind of multiply I guess in a sense because it does bring communities together and it represents, as said before, how multicultural areas can be.

326. I think that we can all like find a place that you can go to or like visit when you feel overwhelmed, stressed.

327. I think that, yeah, we could get closer to our religion and... yeah.. yeah.

328. We can embrace and appreciate.. appreciate different religions.

329. We can, umm, introduce more, umm temples and places of worship and also admire the diversity in the world.

330. I think we can like respect other peoples religions as well as like partaking in our own

331. OK, going back around. Oh no that was the final one. Sorry, Why does this condition exist? I keep missing that one I'm sorry

332. I think it exists to like, give people hope...And like, give them something to pray to so that they feel like grounded.

333. This condition exists because it allows people to go to a place where they can relax and also pray to God.

334. It helps people believe that there is someone out there that's trying to care for you and wants the best for you

335. This condition exists because Newham and East London as a whole, there's loads diversity and religions and stuff, and because of that we need different places of worship

because of course everyone has a different place of worship depending on their religion and stuff.

336. This place exists so that people can go and worship their religion.. like their God or religion.

337. I think places like this exist almost like a sanctuary and a sense of escapism for people to get away from, like, stressful parts of their lives.

338. Think this provides a place for people to worship...And go to who they believe in

339. This condition exists allowing yo...u because it allows people to connect to their spiritual soul.. their spiritual self.

340. Thankyou. And then this is the last picture, [redacted] if you start us off. What do you see here?

341. I see a small lake or a pond in a local park.

342. Looks like some kind of body of water like a...rocky area. And there’s like ducks in the water

343. Yeah I see some ducks along the water and what looks like trees that are dying

344. It looks like a park with like a lake or a river in the middle of it with like ducks and it’s a really nice day

345. Oh, I see... I see yeah a pond with ducks and yeah dying out trees. .And yeah, it seems like a local park.

346. It looks like a park with the river and I think it might be winter time because there's no tree, there's no leaves in the tree.

347. It looks like a lake with ducks and like she said, it looks like it's the winter time because the trees have no leaves.

348. So it looks like a pond and it might be a sunny winter day because there's no leaves, but it's quite bright and there's blue sky and clouds.

349. That’s great, thank you..And then the next question is what is really happening here?

350. What's really happening is the person that took this picture is embracing the natural environment around us.

351. Looks like.... the day, the water, the weather is um being appreciated.

352. It looks like the person is enjoying the scenery.

353. And it looks like the person is just taking in the view.

354. It seems like the person may have come to feed the ducks or to come and just like, come to the park and then they admire how beautiful the water actually looks.

355. It looks like the person is appreciating nature.

356. It looks like the person is there to um, to um, like admire the nature.

357. Yeah, I think people are just like looking at it, feeling calm and peaceful,

358. OK.And then going back this way, how does this picture relate to our lives?

359. I feel like most people will need to like, step back and just admire like our surroundings, because we're always like, really busy and maybe like stressed so I think it's a good technique.

360. [something bangs]

361. [Laughs] that woke everyone up.

362. Most people have visited the rivers and the lakes and fed the ducks.

363. What was the question again?

364. How does.. How does the picture relate to our lives.

365. It can kind of show that our Life can be peaceful. Like this photo
366. It relates to my life because my nephew and my niece always begged me to take them to a park to feed the ducks, and although it’s annoying and I hate doing it it actually gives me a time to go outside and actually like, just get some fresh air with annoying kids.
367. It relates to our lives because there's loads of parks like around us and sometimes we just need to appreciate what we have and like the views that we're surrounded by.
368. I think it relates to our lives because there are lots of parks which kind of are in the area...So it can kind of show like different perspectives of people's local parks.
369. I think that if it is a park then it's easily accessible so a lot of people can come and spend time.
370. So it relates to our lives because it allows us to take a step back from all the buildings and all.. Due to the industrialization and just take in what...just take in nature
371. If we.. should we go back around? Or should we go this way?
372. Yeah, yeah
373. And why does this condition exist?
374. I think it exists to, like, instill calm within in us and just be beautiful.
375. I think this condition exists because it looks like people are looking after it because I don't see any litter on the floor.
376. To provide a peaceful environment for wild animals.
377. So the ducks can get fed [laughter]
378. It exists so that everyone has a place where they can just like, go to relax
379. Think it exists so all people from Like ranging ages of the community can hang out.
380. But like specifically teenagers, it's a free thing to do in London.. So it's a way to have fun.
381. Maybe to walk closer to the water and interact with the ducks
382. It exists for us to take time and just enjoy nature.
383. Okay..And a final question, what can we do about?
384. We can take, we can take some time out of our day and just enjoy the natural environment in the parks
385. Really encourage people to go here to a change of setting.
386. Can promote people to enjoy the nature and scenic places around them.
387. I’m sorry, what's the question?
388. What can we do about?
389. We could try to preserve it and like, keep it as clean as it is so that everyone has a place where they can go whenever they feel like stress or something.
390. We could yeah... Keep it clean, invest more money into it and yeah, go and enjoy it while, while it's still clean
391. Encourage people to just look at the beauty around them and like we would....we would what she said. she said, said.And maybe invest some money into parks like these because it's not always going to be around you.
392. Most people lock themselves up at home.. Promote people to go out and enjoy the nature around us
393. Yeah, like encourage people to go outside and get some fresh air.
394. Brilliant. Great. So that kind of concludes all the pictures So thank you so much for your comments.

395. Like really interesting stuff and definitely things came through like when I was looking at the pictures so much came through during the discussion that I hadn't like seen just by looking at the photos. Obviously, we've used quite like structured approach to these things and I just wanted to use the last kind of closing section of this meeting to see if there was anything else that you wanted to kind of discuss about the pictures. So if I kind of lay them back on the table...If there's anything that you kind o felt like you wanted to say.. if I hold up the first one we spoke about, which was the fountain.Did anyone else have anything else to say about this picture That wasn't kind of spoken about?

396. I wasn't here so [laughter[

397. You weren't here. Is there anything that you have...

398. Yeah, definitely. I feel like it's a.. in such, in so many buildings it's a place to take a break and just enjoy the natural environment, which is really important to our daily lives, yeah.

399. OK, anyone else for this one?

400. Maybe how, like more regenerated areas in East London, are idealized by other people living in the area? So kind of encourage all areas to look like that .

401. It seems too good to be in.East London.

402. [General agreement & laughter] “yeah” “I agree”

403. it looks like due to everyone taking in more like the climate crisis...Like the government and people are ..like councils are trying to show more naturalistic features in...around the busy like buildings and all that.

404. OK. Any other comments on this one? … So next one was the school. Any comments on this that wasn't.. you weren't able to bring up during the...

405. Yeah this is my photo

406. This is what you missed as well.

407. Yeah, so this was.A picture I took. It's basically ...you guys got it right. So it's like a student room, so it's a a study room.This was during break time.I t's really important because it's a picture of like me and my friends and especially during such like a critical time in our lives where we're always so busy.It's nice to just take a step back and communicate with your friends, have a bit of a Laugh and it really changes your state of mind puts you bit of peace...And yeah, that's why I chose this photo. I think its really important to everyone no matter what age group.

408. OK.Anyone else? OK, the sunset one was the third one we discussed.Did anyone else have any other commentsthey couldn't say within the kind of questions we asked

409. Maybe how we need to encourage green spaces from being built on because they do allow people to relax and just offer so many opportunities, like during the summer for teenagers like picnics or just hang out sessions.

410. If you want more nice pictures like this you have to keep thee space clean. Can you imagine if there was litter And you took that picture and it wouldn't work out? [laughter] So if you want these anesthetic really nice pictures, you just have to keep the space clean.

411. Any other final question, final comments on that one. So the next one is the traffic against the pub.

412. Anyone have anything they wanted to say about this? …...No? Okay. Next was one was this one...

413. That was the skyline.

414. I wanna go there today now[laughter]
415. Any other comments?
416. I'm seeing the tall buildings creates like a sense of ambition because I'm sure most of us want to, like, work in big buildings and big offices.
417. Yeah, yeah.I mean, I've got this weird, like, fascination of, like, I really want to go into the HSBC tower.
418. [general agreement] “it’s so big”
419. I always see it and like I just wanna go in there
420. I went to the Barclays one and it’s really, really big so. just looking outside to see to an either bigger one.. Get starstruck
421. So the next one was ...the book and I did really feel for you guys with this one [laughter] because I’ve been there. Any other kind of comments?
422. Don't die for a-levels
423. [Laughs]… don’t die for a-levels
424. Yeah, more like..Just how unrealistic the standards are, [murmur of agreement] especially nowadays, because I understand how the government may want to push us so we can get, like good paying jobs.. But there really aren't that many jobs now because they're being taken up. So, to keep working this hard and then like... like people that I know who literally got A stars and everything are still unemployed to this day.. it doesn't necessarily mean that education is that important to getting finding jobs and just like in general.
425. I think it’s like irony because that topic is about learning about the issues in school.So I’m learning about the issues in school because my school’s telling me to learn about issues with them and stuff.So I always find it a bit ironic that they're teaching us how to like talk about the problem in school, but when we raise an issue in school, they tell us to like, not talk about the issue and stuff.So it's...it seems like we're learning unrealistic stuff that we don't actually need for our future...It's a bit like when we don't get taught about mortgages and taxes even though they're about our live and stuff, they’re gonna help us in the future this sort of stuff doesn’t help anymore.
426. Yeah it’s very niche
427. Yeah
428. Any final comments on that one?
429. Maybe education isn't the only pathway to actually be successful in the future.There's.. nowadays there's so many different ways to actually have a good life...Sort of the traditional ones.
430. Okay. Um, the temple. Any final comments on that one?
431. Kind of like how in East London, every type of culture is really embraced, like there's a place near me where there's a mosque, a church and a synagogue, like pretty much right next to each other. So it's just a really nice way to show how like integrated the communities.
432. It’s so interesting about whoever, like, made it, how they will manage to fit such a huge building in such a small part of like an area and stuff, and how like, like they’ve use such like detailed designs that make it really stand out when you walk past it.
433. Yeah, it's very beautiful. I love this picture, especially with the sky in the background... OK, any final comments on that one? And then the last one, which was the duck pond any final

comments on that one. I’m sensing a no should I put that one down as well? Cool. Brillaint...So we're all done I'm gonna stop recording now so...
